# Differential Gene Expression Reveals Candidate Genes for Drought Stress Response in *Abies alba* (Pinaceae)

**DOI:** 10.1371/journal.pone.0124564

**Published:** 2015-04-29

**Authors:** David Behringer, Heike Zimmermann, Birgit Ziegenhagen, Sascha Liepelt

**Affiliations:** Conservation Biology Group, Philipps-University of Marburg, Germany; National Institute of Plant Genome Research, INDIA

## Abstract

Increasing drought periods as a result of global climate change pose a threat to many tree species by possibly outpacing their adaptive capabilities. Revealing the genetic basis of drought stress response is therefore implemental for future conservation strategies and risk assessment. Access to informative genomic regions is however challenging, especially for conifers, partially due to their large genomes, which puts constraints on the feasibility of whole genome scans. Candidate genes offer a valuable tool to reduce the complexity of the analysis and the amount of sequencing work and costs. For this study we combined an improved drought stress phenotyping of needles via a novel terahertz water monitoring technique with Massive Analysis of cDNA Ends to identify candidate genes for drought stress response in European silver fir (*Abies alba* Mill.). A pooled cDNA library was constructed from the cotyledons of six drought stressed and six well-watered silver fir seedlings, respectively. Differential expression analyses of these libraries revealed 296 candidate genes for drought stress response in silver fir (247 up- and 49 down-regulated) of which a subset was validated by RT-qPCR of the twelve individual cotyledons. A majority of these genes code for currently uncharacterized proteins and hint on new genomic resources to be explored in conifers. Furthermore, we could show that some traditional reference genes from model plant species (*GAPDH* and *eIF4A2*) are not suitable for differential analysis and we propose a new reference gene, *TPC1*, for drought stress expression profiling in needles of conifer seedlings.

## Introduction

Smaller amounts of precipitation and an increase in the occurrence of drought events are being predicted for the Mediterranean region and parts of Central Europe, especially during summer periods [1]. Drought stress poses a major threat to trees by possibly causing hydraulic failure. Facing low water availability, trees react with stomatal closure and reduced photosynthesis while maintaining metabolism. Thus, severe drought periods can ultimately cause forest diebacks by xylem cavitation, carbon starvation and damage by pathogens and insects [2]. While single trees might reveal sufficient plasticity, tree populations can cope with changing climatic conditions either by migration and/or adaptation [3]. Migration via seed dispersal might be increasingly constrained by fragmented landscapes and is generally limited by natural barriers, especially for mountainous species [4]. Adaptation only works via selection on standing genetic variation or newly arisen mutations [5]. Standing variation offers the best chance for rapid adaptation since potentially beneficial alleles might already be numerously present in the population [5].

To assess the adaptive potential of tree populations it is therefore necessary to identify genes that are, among other stress-related traits, involved in the drought stress response and to study their variation in natural populations. Many studies on tree populations today use a candidate gene approach for the following reasons: While genomic resources are readily available for model species such as *Arabidopsis*, rice or maize, for most tree species, and especially conifers, such resources are scarce [6]. Furthermore, the identification of drought stress related genes is a big challenge when working with conifers, due to their large genome sizes [7] which make genome-wide association studies very resource-intensive [8]. Candidate genes are thus used as an alternative for detecting selective signals [9,10]. This approach traditionally involves F_st_ outlier analysis of single-nucleotide polymorphisms (SNPs) within those candidate genes (“bottom-up” approach) [11]. Alternatively, candidate genes allow for a reasonable selection of target genes for association studies (“top-down” approach), especially when handling large genomes. Moreover, drought stress is hard to assess in conifers and the response is a highly quantitative trait.

However, using a novel terahertz spectroscopy setup, it is now possible to continuously measure the water content of multiple plants and thereby precisely monitor the drought stress response [12]. This allows sampling leaves from multiple plants with identical drought stress response and analyzing them with next generation sequencing methods. Thus it is possible to identify those specific genes that are underlying an accurately assessed drought stress phenotype. This approach provides a unique opportunity for detecting and exploring novel candidate genes in non-model species which may not be found annotated from traditional model species. We chose European silver fir (*Abies alba* Mill.) as the model species for our study. *A*. *alba* is an ecologically and economically valuable coniferous tree, which has its main area of distribution in mountainous regions of Central and Southern Europe [13]. Effects of drought were shown to manifest in a reduced growth rate [14–16], reduced photosynthetic activity and stomatal conductance [17,18], crown-damage [19,20] and an increasing susceptibility to damage caused by pathogens or insects [21,22]. A dieback as a response to frequent and severe water shortages can already be observed, e.g. at Mont Ventoux in Southern France [23].

The major goals of our study were (I) the identification of candidate genes for drought stress response in *A*. *alba*, (II) the comparison of drought stress related genes between *A*. *alba* and model organisms to identify conifer-specific genes, (III) the validation of the expression profiles by reverse-transcription quantitative real-time PCR (RT-qPCR) and (IV) the identification of reference genes for RT-qPCR data normalization.

## Materials and Methods

### Plant material and drought stress monitoring

Silver fir seedlings were propagated from seeds of female cones of a single tree in a forest stand near Hagenbach, a Black Forest region of South-Western Germany (the seeds were provided with permission by Hans Lehman from the forestry office Oberharmersbach). Thus, all seedlings used in the experiment were either half-siblings or full-siblings. To establish groups of plants with highly controlled levels of drought stress, a novel terahertz time-domain spectroscopy setup was used in a preliminary study conducted by Born et al. [12]. This allowed the manipulation and monitoring of the individual water status of multiple seedlings by continuously measuring the cotyledons, without inducing other forms of stress. Twelve seedlings were measured this way. While six of them were well-watered, the other six seedlings were not watered until they reached comparable levels of considerable drought stress (for a more detailed account of the drought stress monitoring and its results see Born et al. [12]). At this point, two cotyledons were cut off from each seedling for RNA extraction and immediately stored in liquid nitrogen. Cotyledons were also harvested from the control group of well-watered seedlings at corresponding times of the day.

### RNA extraction

For sequencing, total RNA from every individual needle was extracted using the InviTrap Spin Plant RNA Mini Kit (STRATEC Molecular GmbH, Berlin, Germany). The cotyledons were ground in liquid nitrogen with mortar and pestle in lysis buffer RP and β-Mercaptoethanol. Half of each lysate was used for RNA extraction by GenXPro GmbH (Frankfurt am Main, Germany) while the rest was stored at -80°C for RT-qPCR validation. To remove genomic DNA contaminants the samples were treated “off-column” with Baseline-ZeroTM DNase (Epicentre/Biozym, Hessisch Oldendorf Germany) and subsequently purified using RNA Clean & ConcentratorTM-5 Kit (Zymo Research Europe, Freiburg Germany). RNA samples for RT-qPCR validation were immediately stored in a deep freezer at -80°C. RNA concentration and purity were measured via ratios of optical density (OD_260/280_, OD_260/230_) using NanoDrop 1000 spectrophotometer (PEQLAB Biotechnologie GmbH, Erlangen Germany). The absence of DNA contamination was confirmed after performing a PCR using a primer pair which targets the nuclear microsatellite marker NFH15 (GenBank Accession Number: AY966492, [24]) at an annealing temperature of 57°C. Integrity was assessed using gel-electrophoresis. Complementary DNA (cDNA) was synthesized using the Maxima First Strand cDNA Synthesis Kit for RT-qPCR (ThermoScientific, Schwerte Germany). The cDNA samples were immediately stored in aliquots at -80°C. All kits were applied according to the manufacturer’s protocol. Any modifications are explicitly described.

### Transcriptome sequencing

Prior to synthesizing cDNA, the extracted mRNA from the drought stressed and the well-watered seedlings was pooled, respectively. From each pool a cDNA library was constructed targeting sequences near the cDNA 3’-ends. This Massive Analysis of cDNA Ends (MACE) was conducted by GenXPro GmbH as described in Kahl *et al*. [25]. The 5’-ends of 50–500 bp long fragments were sequenced (single-read) using the Illumina HiSeq 2000 platform (Illumina Inc., San Diego, CA, USA), generating 100 bp long tags. Illumina’s HiSeq Control Software v. 2.0.5 was used for sequencing, RTA v. 1.17.20.0 for real time analysis and CASAVA v. 1.8.2 (Consensus Assessment of Sequence and Variation) for base calling and demultiplexing. To prevent PCR-biased quantification, GenXPro’s “TrueQuant” method was applied, thereby eliminating PCR-based copies from the dataset. For this purpose, unique oligonucleotides were ligated to each tag prior to PCR, making it possible to identify and eliminate PCR copies with identical barcode-tag-combinations [26,27].

### Assembly, annotation and gene expression profiling

After sequencing, the tags were assembled and annotated using the TIGR Plant Transcript Assemblies database (http://plantta.jcvi.org/). Not annotated tags were assembled and subsequently blasted (BLASTx) against the Swiss-Prot and TrEMBLE databases (http://www.uniprot.org/). A differential expression analysis was conducted using the MA-plot based method with random sampling model (MARS) of the DEGseq R package [28]. Prior to this analysis the libraries were normalized according to their respective size by dividing each tag frequency through the sum of the total tags and multiplied by 10^6^ (tags per million). For multiple testing corrections a *p*-value-threshold of 1e-10 for significantly differentially expressed (DE) transcripts was set. Following, the enrichment of each gene ontology (GO) term was tested using Fisher’s exact test (two-tailed) [29]. Additionally, we analyzed the MACE results using the R packages DESeq [30] with a single estimated dispersion condition, a size factor normalization and an FDR (false discovery rate) threshold of *q* < 0.1 as well as NOISeq [31] with simulated technical replicates (NOISeq-sim), a trimmed mean of M-values normalization and a threshold of *q* = 0.9.

### RT-qPCR validation

The gene expression of a small number of genes was assessed in each individual seedling by RT-qPCR using relative quantification according to the MIQE criteria (Minimum Information for the Publication of Quantitative Real-Time PCR Experiments) [32].

The MACE dataset was first searched for DE transcripts with log_2_ fold changes higher than 3 (for up-regulated transcripts) or lower than -3 (for down-regulated transcripts) since they were most likely responsive to dehydration. To minimize the rate of false positives introduced by rare transcripts, a threshold of at least 50 different tags with match in sense orientation (5’-3’) to a database entry was set. Genes for validation were selected from this filtered subset of DE transcripts that were significantly assigned (enrichment-*p*-value < 1e-10) to the GO terms response to water stimulus (GO:0009415), response to water deprivation (GO:0009414) and response to osmotic stress (GO:0006970). Furthermore, genes were selected from the subset of filtered DE transcripts with the ten highest and ten lowest fold changes that were significantly assigned to the GO domain biological process (GO:0008150). Primer pairs (Metabion, Martinsried, Germany) were designed based on the assembled MACE tag sequences for each selected gene using Primer3 v. 4.0.0 (http://bioinfo.ut.ee/primer3/) with default parameters for a product size of 60 bp to 150 bp and an optimum annealing temperature of 60°C. Primer pairs were considered specific when (1) there was no amplicon present in genomic DNA samples, (2) the first derivative of the corresponding melting curves resulted in a single peak, (3) gel-electrophoresis showed one product with the expected size and (4) the amplicon-sequence was identical with the target sequence which was verified by re-sequencing of the PCR-products using the Macrogen Europe Laboratory sequencing service (Amsterdam, The Netherlands).

To select adequate reference genes for the normalization of the RT-qPCR data two different approaches were used. First, by searching the literature for conifer gene expression studies, traditionally used reference genes were identified. Second, the MACE-dataset was searched for sequence tags which were neither up- nor down-regulated (*p*-value > 0.99, log_2_ fold change: -0.005 to 0.005), and had a minimum amount of ten sequence tags. The potential reference genes were tested for their expression stability among the drought stressed and well-watered seedlings using geNorm [33] and Normfinder [34]. Both algorithms were implemented in GenEx v. 5.4.4.119 (MultiD Analyses AB, Göteborg Sweden) which also provided the expected accumulated standard deviation to assess the optimal number of reference genes to be included for the most precise data normalization [35]. Real-time PCR was performed on the Roche LightCycler 480 II System (Roche Diagnostics, Mannheim, Germany) using the sample maximization method with samples in triplicates at an optimized and standardized temperature and cycle program (Table S1 in S1 File) in which only the annealing temperatures were varied according to the optimum of the primer pairs (Table S2 in S1 File). Each PCR reaction was performed with KAPA SYBR Fast Universal Master Mix (Peqlab, Erlangen, Germany).

After the RT-qPCR the quantification cycles (Cq) were determined using the second derivative maximum method implemented in the Roche LightCycler 480 Instrument Software v. 1.5.0. The gene expression ratio was calculated using the Pair Wise Fixed Reallocation Randomization Test implemented in the Relative Expression Software Tool-384 v. 1 (REST) using 5000 iterations [36]. The ratio was corrected for the amplification efficiencies which were calculated according to Liu & Saint [37]. The intra- and inter-assay variations were assessed by calculating the coefficient of variance as the standard deviation relative to the mean of the Cq-values. Therefore, thirty replicates of the same cDNA sample were used to amplify *GAPDH* in three separate qPCR runs (each with ten of the replicates) on three different days. The coefficient of variance was not supposed to exceed four percent on the Cq basis [38].

## Results

### MACE libraries

After sequencing, the MACE method yielded two libraries containing, in total, 15.4 million tags with 6.2 million tags for the drought stressed pool and 9.2 million tags for the well-watered pool (Table 1).

**Table 1:** Characteristics of the MACE libraries constructed from the drought stressed and the well-watered seedlings.

	Tags (total)	Tags (unique)	Drought stressed	Well-watered
Hits (S+AS)	14,162,592	6,435,157	5,664,178	8,498,414
No hit	1,275,004	1,029,367	542,833	732,171
Total	15,437,596	7,464,524	6,207,011	9,230,585

Shown is the amount of total tags analyzed for the library, the amount of unique tags and the amount of tags for each treatment pool (drought stressed and well-watered).

S: sense direction; AS: antisense direction.

**Fig 1:**
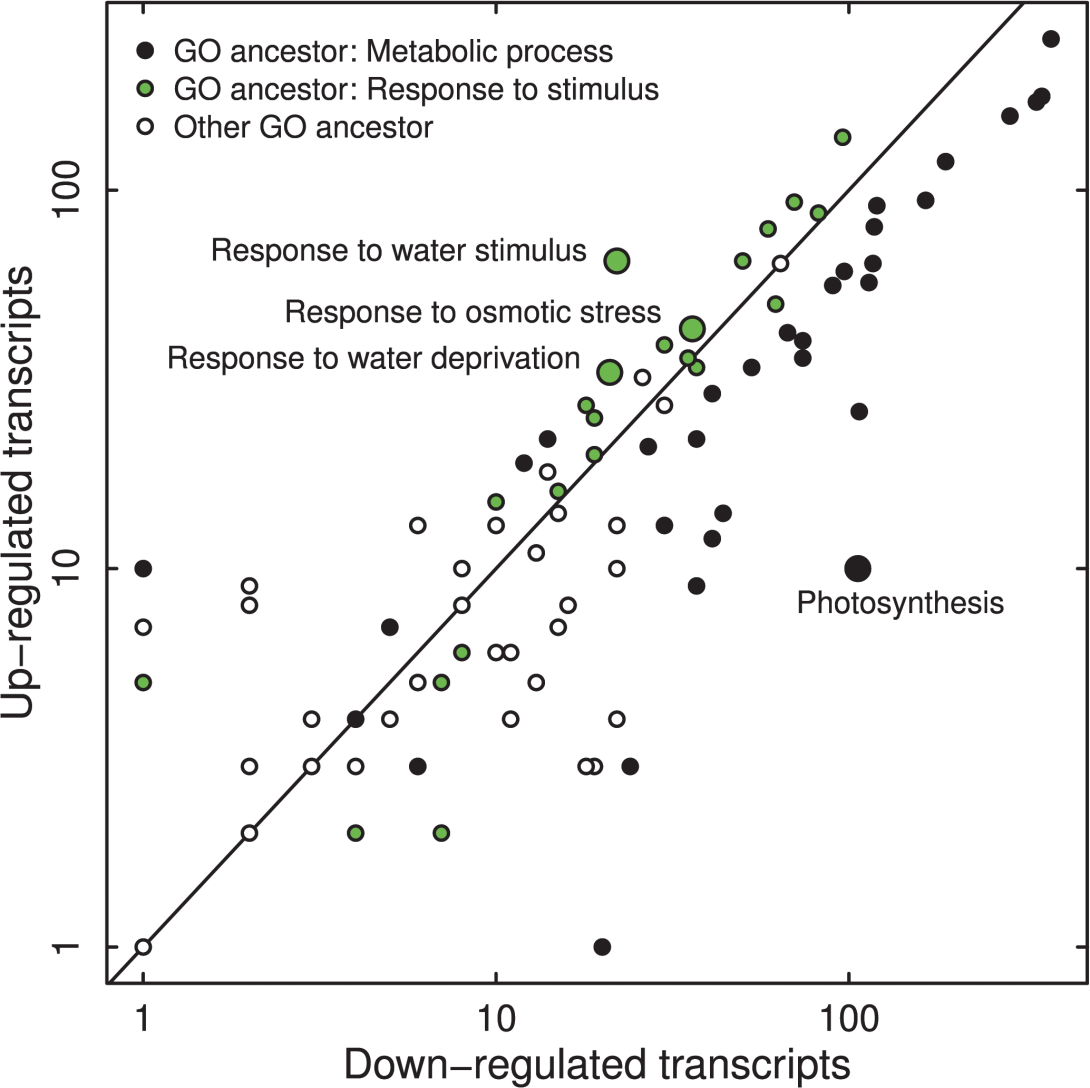
Log-log plot of up- and down-regulated transcripts in response to drought stress on GO-level 4 in silver fir seedlings. Transcripts are differentiated by their GO ancestor: metabolic process (GO:0008152), response to stimulus (GO:0050896) or other ancestor. Most obvious GO terms associated with drought stress, as well as photosynthesis, are highlighted and labeled specifically.

Annotation of the tags resulted in a total of 65,535 transcripts, which were assigned to the three main gene ontology (GO) domains: molecular function (GO:0003674) contained 38,745 transcripts, cellular component (GO:0005575) 39,776 and biological process (GO:0008150) 37,140. Since this analysis aimed to find candidate genes associated with drought stress response, transcripts assigned to the GO domain biological process were most interesting. Within this domain, GO terms associated with metabolic processes were most enriched. In response to drought stress these GO terms were mostly down-regulated, as was methylation (GO:0032259) and photosynthesis (GO:0015979) (Fig 1 and Table S3 & S4 in S1 File). In contrast, GO terms associated with stimuli and stress were generally up-regulated, especially terms most obviously linked to drought stress, namely response to water stimulus, response to water deprivation and response to osmotic stress.

### Differential gene expression analyses

**Fig 2:**
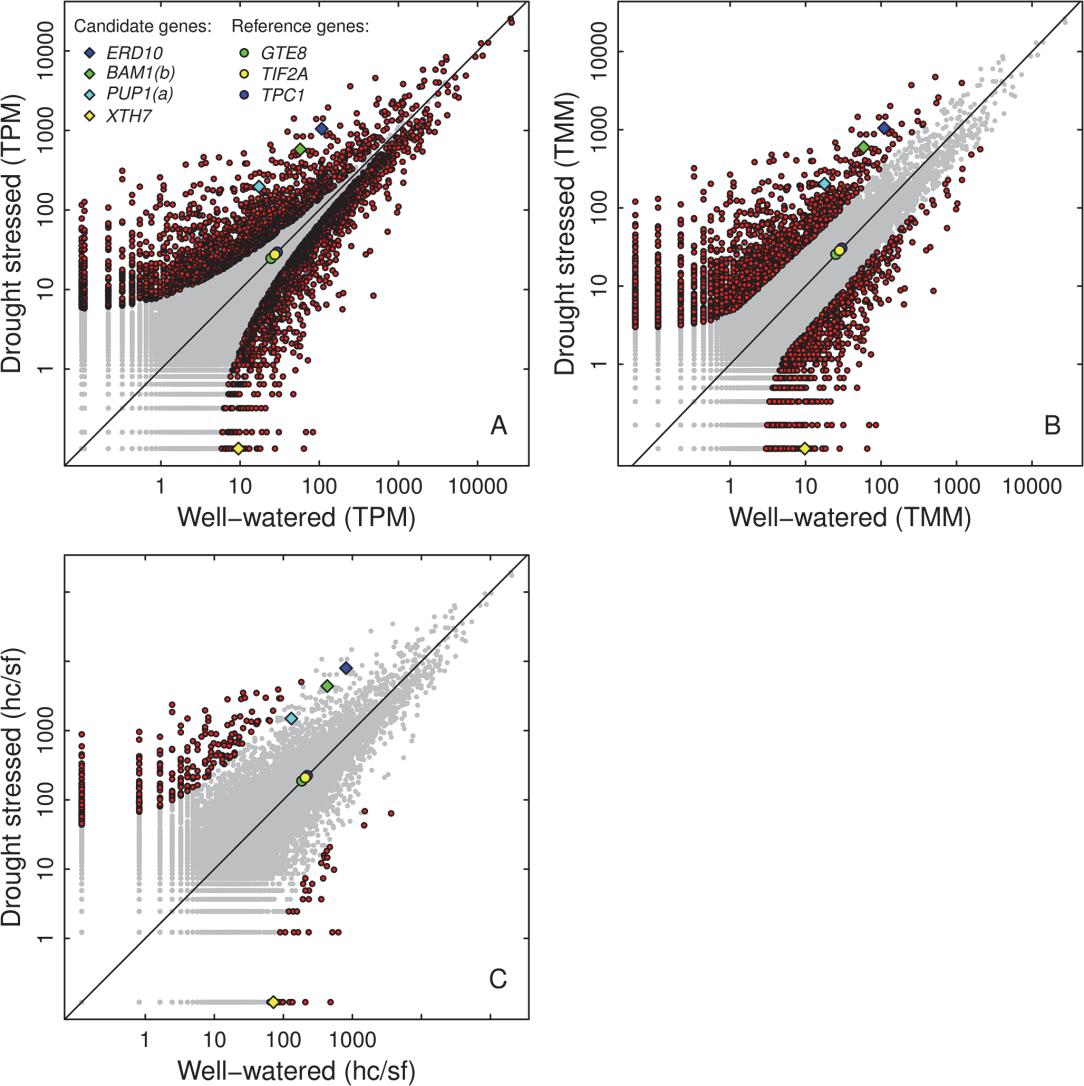
Scatter plots of the MACE results and subsequent analyses of differential expression for DEGseq (A), NOISeq (B) and DESeq (C). Each plot contains all identified transcripts (grey dots), as well as the analysis-specific DE transcripts (red dots). Further, the candidate genes validated via RT-qPCR are shown, as well as the corresponding stably expressed reference genes. The x- and y-axis give the transcript count in the well-watered and the drought stressed pool, respectively. Counts are normalized differently for the three analyses: tags per million (TPM) for DEGseq, trimmed mean of M-values (TMM) for NOISeq and hitcount/size factor (hc/fc) for DESeq. Transcripts falling on the straight line (90° bisecting line) are equally expressed in both pools. Transcripts above the line are up-regulated in response to drought stress, while those below the line are down-regulated.

**Fig 3:**
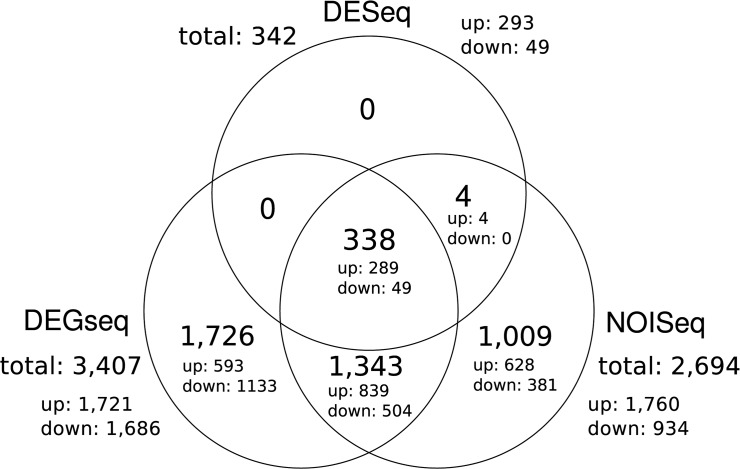
Venn diagram of the overlapping DE transcripts from the DEGseq, DESeq and NOISeq analyses. The amount of up- and down-regulated transcripts is given for each segment.

The DEGseq analysis resulted in a total of 3,407 significantly DE transcripts (*p* < 1e-10) between the drought stressed and the well-watered pool (Fig 2A). The NOISeq (*q* = 0.9, Fig 2B) and DESeq (*q* < 0.1, Fig 2C) analyses yielded 2,694 and 342 DE transcripts, respectively. DEGseq uniquely identified 1,726 transcript and NOISeq 1009 transcripts, while DESeq shared all identified transcripts with either NOISeq or both DEGseq and NOISeq (Fig 3).

### RT-qPCR validation and candidate gene selection

After filtering by fold change and the minimum number of different sense tags (≥ 50), out of the 3,407 DE transcripts identified by DEGseq, 832 transcripts could be listed (Table 2, FASTA files of all 832 transcripts available in S2 File). From the subset of filtered DE transcripts with a significant assignment to the GO terms response to water stimulus, response to water deprivation and response to osmotic stress and the top ten up- and down-regulated transcripts significantly assigned to biological process, 29 different up-regulated (Table S5, S6 & S8 in S1 File) and 14 different down-regulated (Table S7 & S8 in S1 File) genes for validation could be listed (Table 3). All these selected genes were represented in the NOISeq results, while DESeq did not identify eight up-regulated and seven down-regulated genes as DE transcripts.

**Table 2:** Differentially expressed transcripts resulting from the MACE and DEGseq analyses, filtered by log_2_ fold change and minimum different sense tags (≥ 50).

	Up-regulated	Down-regulated	Total
**With database hit** ^**a**^	330	125	455
Assigned to biological process	217	77	294
Not assigned to biological process	113	48	161
**Without database hit**	253	124	377
**Total**	583	249	832

Shown is the amount of transcripts with and without database hit and significant assignment to the GO domain biological process (enrichment-*p*-value < 1e-10).

^a^‘Database hit’ refers to transcripts with database accession number or similarity to a UniProt Reference Cluster (UniRef) sequence.

**Table 3:** Genes for validation derived from the DEGseq analysis; sorted by log_2_ fold change in descending order.

Gene name	Abbreviation	Protein name	Possible isoform	Accession number	Source organism	Fold change
*-*	-	Polyphenol oxidase A1	*-*	Q06215	*Vicia faba*	10.61
*Os01g0656200*	-	Probable protein phosphatase 2C 8	*-*	Q5SN75	*Oryza sativa subsp*. *japonica*	9.55
*-*	-	Glucan endo-1,3-beta-glucosidase, acidic isoform	-	P49237	*Zea mays*	9.24
*At4g33300*	-	Probable disease resistance protein At4g33300	*-*	Q9SZA7	*Arabidopsis thaliana*	8.63
*dhn2*	-	Dehydrin 2 (fragment)	*-*	E1A556	*Pinus pinaster*	8.56
*-*	*PUP2*	Putative uncharacterized protein 2	*-*	A9NPH4	*Picea sitchensis*	8.50
*CXE15 or CXE2*	-	Probable carboxylesterase 15 or 2	*-*	Q9FG13 or Q9SX78	*A*. *thaliana*	8.42
*Cht8*	-	Chitinase 8	*-*	Q7XCK6	*O*. *sativa subsp*. *japonica*	8.37
*GOLS2*	-	Galactinol synthase 2	*b*	Q9FXB2	*A*. *thaliana*	8.27
*GSTU19*	-	Glutathione S-transferase U19	*-*	Q9ZRW8	*A*. *thaliana*	6.82
*BAM1*	-	Beta amylase 1, chloroplastic	*a*	Q9LIR6	*A*. *thaliana*	6.74
*TIP1-1*	-	Aquaporin TIP1-1	*-*	P25818	*A*. *thaliana*	5.75
*LTI6B*	-	Hydrophobic protein LTI6B	*a*	Q0DKW8	*O*. *sativa subsp*. *japonica*	5.63
*STP13*	-	Sugar transport protein 13	*-*	Q94AZ2	*A*. *thaliana*	5.52
*RHA2A*	-	E3 ubiquitin-protein ligase RHA2A	*a*	Q9ZT50	*A*. *thaliana*	5.36
*GOLS1*	-	Galactinol synthase 1	-	Q947G8	*Solanum lycopersicum*	4.94
*LEA14-A* ^*a*^	-	LEA protein Lea14-A	*-*	P46518	*Gossypium hirsutum*	4.88
*GOLS2*	-	Galactinol synthase 2	*a*	C7G304	*S*. *lycopersicum*	4.60
*LTI6B*	-	Hydrophobic protein LTI6B	*b*	Q0DKW8	*O*. *sativa subsp*. *japonica*	4.52
*RCI2A* ^*a*^	-	Hydrophobic protein RCI2A	*-*	Q9ZNQ7	*A*. *thaliana*	4.02
*CIPK17* ^*a*^	-	CBL-interacting protein kinase 17	*-*	Q75L42	*O*. *sativa subsp*. *japonica*	3.73
*-*	*PUP1*	Putative uncharacterized protein 1	*b*	A9NLY4	*P*. *sitchensis*	3.55
*Zlp* ^*a*^	-	Zeamatin	*-*	P33679	*Z*. *mays*	3.51
*-*	PUP1*	Putative uncharacterized protein 1	*a*	A9NLY4	*P*. *sitchensis*	3.50
*BAM* ^*a*^ *	-	Beta amylase 1, chloroplastic	*b*	Q9LIR6	*A*. *thaliana*	3.33
*ERD10* ^*a*^ *	-	Dehydrin ERD10	*-*	P42759	*A*. *thaliana*	3.29
*KCS11*	-	3-ketoacyl-CoA synthase 11	*a*	O48780	*A*. *thaliana*	3.20
*-*	-	LEA protein^*a*^	*-*	P21298	*Raphanus sativus*	3.16
*MGL* ^*a*^	-	Methionine gamma-lyase	*-*	Q9SGU9	*A*. *thaliana*	3.00
*TUBB8* ^*a*^	-	Tubulin beta-8 chain	*-*	P29516	*A*. *thaliana*	-3.04
*UGT74E2* ^*a*^	-	UDP-glycosyltransferase 74E2	*-*	Q9SYK9	*A*. *thaliana*	-3.16
*XTH6* ^*a*^	-	Probable xyloglucan endotransglucosylase/hydrolase protein 6	*-*	Q8LF99	*A*. *thaliana*	-4.25
*KCS11* ^*a*^	-	3-ketoacyl-CoA synthase 11	*b*	O48780	*A*. *thaliana*	-5.69
*GPPS3* ^*a*^	-	Geranyl diphosphate synthase	*-*	Q8LKJ1	*Abies grandis*	-5.92
*-*	*PUP5* ^*a*^	Putative uncharacterized protein 5	*-*	B8LN73	*P*. *sitchensis*	-5.94
*RHA2A*	-	E3 ubiquitin-protein ligase RHA2A	*b*	Q9ZT50	*A*. *thaliana*	-6.02
*-*	*PUP4* ^*a*^	Putative uncharacterized protein 4	*-*	C0PT89	*P*. *sitchensis*	-7.27
XTH7*	-	Probable xyloglucan endotransglucosylase/hydrolase protein 7	*-*	Q8LER3	*A*. *thaliana*	-7.57
*COL6*	-	Zinc finger protein CONSTANS-LIKE 6	*-*	Q8LG76	*A*. *thaliana*	-7.80
*-*	-	Patatin-like protein 3	*-*	B6TPQ5	*Z*. *mays*	-8.02
*VTE4*	-	Tocopherol O-methyltransferase	*-*	Q9ZSK1	*A*. *thaliana*	-8.72
*INR1*	-	Inducible nitrate reductase [NADH] 1	*-*	P54233	*Glycine max*	-9.01
*-*	*PUP3*	Putative uncharacterized protein 3	*-*	F6HZZ7	*Vitis vinifera*	-10.32

Gene names according to UniProt Protein Knowledgebase (http://www.uniprot.org/) with the corresponding database accession number.

- No gene name available or abbreviation assigned;

* Validated via RT-qPCR;

^*a*^ Not identified as DE by DESeq.

The literature search revealed twelve reference genes from published conifer studies (Table S9 in S1 File). Profiling the MACE dataset revealed three potential reference genes: the global transcription factor group E8 (*GTE8*), the transcription initiation factor 2A (*TIF2A*) and the two pore calcium channel protein 1 (*TPC1*). All three exhibited medium abundance levels and were therefore optimal candidates as reference genes. Out of the 15 potential reference genes, four (*GAPDH*, *TPC1*, 18S rRNA and *elF4A2*) specifically amplified their target in the RT-qPCR. The expression stability of these genes was tested using Normfinder [34] and geNorm [33]. Both algorithms identified *TPC1* and 18S rRNA as the most stable reference genes (Table S10 in S1 File). Furthermore, their combination exhibited the lowest accumulated standard deviation. Thus, *TPC1* and 18S rRNA were both applied for normalization in REST.

Out of the 50 tested primer pairs for validation (Table S2 in S1 File), four could meet our conservative criteria and were specifically amplifying their targets during the qPCR: *ERD10*, *BAM1(b)*, *XTH7* and *PUP1(a)* (Table S11 in S1 File). The calculated gene expression ratios were similar to those obtained by the MACE method (Table 4). The assay precision was assessed by calculating the intra-assay variation (repeatability) and inter-assay variation (reproducibility) based on Cq-values. The intra-assay coefficient of variance ranged between 0.8% and 1.08% while the inter-assay coefficient of variance was 0.92%.

**Table 4:** Log_2_ fold changes of the four specifically amplified genes for validation resulting from the MACE analysis and RT-qPCR (calculated using REST with correction of amplification efficiencies).

Gene name	log_2_ fold change MACE	log_2_ fold change RT-qPCR ± SE	Pair-wise fixed reallocation randomization test *p*-value
*BAM1(b)*	3.33	2.50 ± 0.97	0.0052
*ERD10*	3.29	2.91 ± 1.36	0.0016
*PUP1(a)*	3.50	3.95 ± 2.60	0.0016
*XTH7*	-7.57	-3.83 ± 4.958	0.001

SE = standard error.

From the 338 transcripts identified unanimously by all Seq-analyses (Fig 1), 296 remained after filtering by minimum different sense tags (Table 5, FASTA files of all 296 transcripts available in S3 File). Almost half of these transcripts (~45%) were unknown and many of the transcripts with a database hit (~38% of the remaining ~55%) were not yet properly assigned.

**Table 5:** Properties of the sense-tag-filtered consensus transcripts identified by the three different Seq-analyses.

	Up-regulated	Down-regulated	Total
**With database hit** ^**a**^	140	22	162
PUPs (*Picea sitchensis*)	43	5	48
PUPs (other species)	2	1	3
UPs	7	1	8
Similarity to UniRef sequence	3	0	3
**Without database hit**	107	27	134
**Total**	247	49	296

PUP: Putative uncharacterized protein; UP: Uncharacterized protein.

^a^‘Database hit’ refers to transcripts with database accession number or similarity to a UniProt Reference Cluster (UniRef) sequence.

## Discussion

Analyzing the adaptive potential of plant species to drought stress is of crucial importance in the context of rapid climate change. For species with large genomes, such as conifers, where genomic resources are scarce, it is often necessary to reduce the pool of target genes to an affordable size. Combining a new water monitoring setup with the MACE technique we were able to link a standardized phenotypic response to specific genes and thereby identify novel candidate genes for drought stress response in *A*. *alba*.

For our approach, we pooled RNA from individual seedlings for each treatment group and subsequently analyzed both pools via transcriptome sequencing. To exclude bias by individual expression patterns we applied RT-qPCR to validate the differential expression for a subset of genes known to be involved in the drought stress response of model plant species. Among these genes, only *UGT74E2* displayed a gene-regulation pattern that clearly differed from expectation according to the respective literature. In *Arabidopsis*, an ectopic over-expression of *UGT74E2* increased the tolerance to salinity and drought stress and reduced the plants’ water loss [39]. Therefore, one would assume an up-regulation in response to drought stress. However, in silver fir the opposite was the case. Hence, *UGT74E2* might play a different physiological role in the phylogenetically distant silver fir, compared to *Arabidopsis*. Further research regarding the function of *UGT74E2* in other conifer taxa are necessary to offer an explanation for the different expression in response to drought stress.

The up-regulation of *PUP1(a)*, *BAM1(b)* and *ERD10* based on the MACE technique and subsequent DEGseq analysis could be verified by the RT-qPCR. Individual differences among the seedlings were expected but proved to be low for *BAM1(b)* and *ERD10* and moderate in the case of *PUP1(a)*. Though varying to a larger extent, the overall down-regulation of *XTH7* could be affirmed. However, DEGseq predicted a much higher gene expression ratio than observed with RT-qPCR. Individual differences were more pronounced for *XTH7* and may partially be attributed to PCR inhibition and/or stochastic cDNA template variation.

In order to select adequate reference genes for the RT-qPCR, we tested traditional reference genes which were previously used in other conifer gene expression studies. Depending on the treatment, ontogenetic stage or the tissue under investigation some of them showed expression stability [40–42], even though in other studies [33,40,42,43] these genes showed significant variability in expression patterns. Here we tested *GAPDH*, *elF4A2* and 18S rRNA, with only 18S rRNA showing expression stability across all individual seedlings. Since the expression of *GAPDH* and *eIF4A2* varied among the drought stressed and well-watered seedlings, these genes were not suitable as internal controls. However, 18S rRNA should not be used as the only reference gene for normalization due to the possible mismatch of rRNA and mRNA abundance [44]. Fortunately, the selection of reference genes based on the MACE results proved to be successful. We could verify the expression stability of *TPC1* for all individual seedlings using RT-qPCR. In *Arabidopsis* and rice *TPC1* is known to be ubiquitously expressed across several tissues [45,46]. However, Wang *et al*. [47] identified a *TPC1* homologous gene which was induced in *Triticum aestivum* as a response to high salinity, polyethylene glycol (PEG), low temperature and abscisic acid treatment. They suggested an important role of *TPC1* in the stomatal closure and abiotic stress response of *T*. *aestivum*. On the one hand this implies the necessity for further investigation on the expression stability of *TPC1* as a potential reference gene when investigating drought stress response. On the other hand the *TPC1* homologue might be involved in the osmotic rather than the drought stress response. Since the expression stability of *TPC1* was only tested in cotyledons, future studies need to address other tissues such as roots, as well as different age stages of needles.

Our study design lacked biological replicates, which is a serious limitation but was dampened by pooling the samples for the two libraries. Pooling samples for RNA-Seq analysis has proven to be a reliable method for estimating gene expression, especially for genes exhibiting high expression levels [48]. Furthermore, the THz measurements were highly precise which ensured that the pools had very homogenous stress levels [12]. It is also notable that the THz approach enabled us to measure a stress response solely induced by water deprivation. Other approaches, such as PEG treatment, might largely lead to the differential expression of genes involved in osmotic stress response rather than in the response to water shortage. Since the individuals in our study were pooled in two treatment groups we could not estimate the biological variation within those groups. As stated in other studies facing the same problem [49–51], the results must be taken cautiously and the candidates should be further examined. Nonetheless, in order to analyze our data, we chose a conservative approach. Therefore, apart from DEGseq, we additionally employed DEseq and NOISeq. All three analyses showed different results as was expected according to comparative studies of the used methods [52,53]. DESeq is generally more conservative, while DEGseq and NOISeq are more aggressive but prone to false positives. However, DESeq did not identify three of the four genes verified by RT-qPCR as DE transcripts (Fig 2). Since the genes for validation were selected by very strict criteria and the RT-qPCR was conducted on the individual seedlings and not on pooled plant material, we conclude that the DEGseq and NOISeq results likely include a relatively high amount of false positives, while the 43 genes for validation (Table 3) should be correctly defined as DE transcripts. Since these genes were representatives of the group of 832 filtered DE transcripts, we define the whole set as potential candidate genes for drought stress response for further studies. However, the most conservative selection would only include the 296 filtered consensus transcripts.

Some of these genes or close variants were previously identified or used as candidates in other studies regarding drought stress response in conifers. For example, xyloglucan endotransglycosylase/hydrolase (*AoXET1*) was down-regulated in needles and stems of *Pinus pinaster* seedlings in response to drought stress [54]. A glutathione S-transferase and chitinases (*cht1* and *cht2*) were up-regulated in response to drought- and pathogen-related-stress in roots and shoots of six-week-old seedlings of *Picea abies* [55]. Velasco-Conde et al. [56] measured the expression pattern of several dehydrins (*dhn1*, *dhn2*, *dhn3*, *dhn7*, *dhn9* and a *dhn-*like protein) in needles of 3-year-old cuttings of drought-sensitive and drought-resistant genotypes of *P*. *pinaster*. Only *dhn3* and *dhn4* showed an involvement in drought resistance between genotypes, while *dhn2* was consistently down-regulated in response to drought stress. Our results suggest that *dhn2* might play a different role in drought stress response in *A*. *alba*, since it was significantly up-regulated. Dehydrins (*dhn1* and *dhn2*), aquaporin (*aquaMIP*) and early responsive to dehydration 3 (*erd3*) were used as candidate genes for drought stress response in megagametophytes of *Pinus taeda* [57]. Similarly, a putative glucan-endo-1,3-beta-glucosidase precursor, dehydrins (*dhn1* and *dhn2*) and *erd3* were used as candidates for outlier analyses in megagametophytes of *P*. *pinaster* [58]. Chitinase 4 and a putative LEA protein were identified as drought stress responsive in needles, stems and roots of *P*. *pinaster* [59] and a glutathione S-transferase in needles of adult *Pinus halepensis* trees [50].

To our knowledge, no studies exist, which aimed to identify adaptive genes for drought stress response in any member of the genus *Abies*. However, such an analysis would surely benefit from genus-specific candidate genes. Here we present a selection of 296 genes that contains previously identified candidates but predominantly adds to the possible selection of candidates for future studies. Many of these genes are linked by their biological function to a specific response to water deprivation. For example, 3-ketoacyl-CoA synthase 11 belongs to the group of “very long chain fatty acids”, which are required for wax synthesis [60]. The leaf cuticle is protected by the wax layer against non-stomatal water loss, which could explain its up-regulation. Also involved in water management are aquaporins, which are expressed very variably in response to drought stress [61]. Tonoplast intrinsic proteins (TIP1s) are usually found in the lytic vacuole membrane [62]. Hence, *TIP1-1* is probably up-regulated during drought periods to allow better access to the water stored in the vacuole. Chloroplastic beta-amylase 1 is involved in the breakdown of leaf starch [63]. During daytime, plants store glucose as starch in chloroplasts and access this energy during nighttimes via starch breakdown. Up-regulation of *BAM1* is most likely a response to the down-regulation of metabolic processes and especially photosynthesis during drought periods (Fig 1). Analogous to “regular” nighttimes, metabolism with reduced photosynthesis can only be maintained by breaking down the energy storage, e.g. starch. Accordingly, sugar transport proteins, which transport hexoses through cellular membranes, are necessary for metabolism but may also play a role in distributing osmolytes throughout the plant. The down-regulation of *XTH6* and *XTH7* indicates limited cell growth during drought periods, since xyloglucan endotransglucosylase/hydrolases are involved in cell enlargement and restructuring [64]. Protein kinases add phosphate groups to a substrate, while protein phosphatases remove them, thus either activating or deactivating enzymes [65]. Both protein kinases and phosphatases are key factors in signal transduction as response to drought stress, by regulating enzyme activity. E3 ubiquitin-protein ligase RHA2A functions in an ABA-mediated signaling pathway during early seedling development, positively regulating the plants response to osmotic stress [66]. The fact that *RHA2A* is part of the subset of both up- and down-regulated candidate genes highlights the necessity for a distinction between possibly different protein isoforms for all genes. Late embryogenesis abundant (LEA) proteins play a protective role against desiccation-damage during drought periods, presumably by suppressing protein aggregation [67]. Dehydrins were initially categorized as “Group II LEA proteins” and indeed protect plant cells from desiccation-damage but are also involved in pathogen resistance [68]. Galactinol synthases are induced by drought, cold and ABA [69] and are interesting from the perspective of adaptation. Taji et al. [70] found that genes encoding galactinol synthase in transgenic *Arabidopsis* improved drought stress tolerance, which might be attributable to the role of galactinol synthase in the biosynthesis of “raffinose family oligosaccharides”. The resulting accumulation of galactinol and raffinose may enhance drought stress tolerance via osmoprotection. Chitinase 8, the acidic isoform of glucan endo-1,3-beta-glucosidase and zeamatin are all involved in pathogen defense response, mainly against fungi [71–73] and in the case of probable disease resistance protein At4g33300 against bacteria [74]. Reason for the up-regulation of pathogen-resistance genes in silver fir in response to drought might be that forest trees are especially prone to drought-disease interactions, mainly involving fungi [21]. Glutathione S-transferases are most notably detoxification enzymes with many other, still unknown, involvements hypothesized in plant stress response [75]. As such, they are classified as early responsive to dehydration (ERD), i.e. genes that are activated swiftly in response to drought stress, a group that also contains dehydrins [76].

A group of gene products that stands out are the putative uncharacterized proteins (*PUPs*) inferred from the transcriptome sequencing of *Picea sitchensis* [77]. *PUP1* belongs to the dehydrin family, *PUP2* to the NAC domain and *PUP4* as well as *PUP5* to the family of UDP glycosyltransferases (UGT), according to the *UniProt Protein Knowledgebase* (http://www.uniprot.org/). All *PUPs* identified in this study seem to play an important role in the drought stress response of silver fir and should be focused on in further studies. Correspondingly, further research should focus on the DE transcripts that did not have a database hit, since these genes are not yet described for any plant species and are most likely involved in drought stress response. As such, they might possibly be specific to the Pinaceae family or conifers in general.

In conclusion our study provides first insights into the drought stress response of *A*. *alba* at the transcriptome level and offers a set of candidate genes for use in future studies. The majority of these candidates are yet unknown or lack a proper assignment and add to the growing genomic resources available for non-model conifer species. Such resources will be increasingly important for investigating the adaptive potential of long-lived organisms such as trees in the face of rapid climate change.

## Supporting Information

S1 File File containing all supporting tables. (DOCX)

S2 File FASTA files of all 832 DE transcripts identified by DEGseq and filtered by different sense tags (≥ 50) and log_2_ fold change (< -3 or > 3). (GZ)

S3 File FASTA files of all 296 DE transcripts identified unanimously by DEGseq, DESeq and NOISeq and filtered by different sense tags (≥ 50). (GZ)

## References

[1] IPCC. Climate Change 2007: Working Group I: The Physical Basis. Contribution to the Fourth Assessment Report of the Intergovernmental Panel on Climate Change. Chapter 11: Regional Climate Projections. 2007.

[2] Allen CD, Macalady AK, Chenchouni H, Bachelet D, McDowell N, Vennetier M, et al A global overview of drought and heat-induced tree mortality reveals emerging climate change risks for forests. Forest Ecology and Management. 2010;259:660–684. 10.1016/j.foreco.2009.09.001

[3] Aitken SN, Yeaman S, Holliday JA, Wang T, Curtis-McLane S. Adaptation, migration or extirpation: climate change outcomes for tree populations. Evolutionary Applications. 2008;1:95–111. 10.1111/j.1752-4571.2007.00013.x 25567494 PMC3352395

[4] Pearson RG, Dawson TP. Predicting the impacts of climate change on the distribution of species: are bioclimate envelope models useful? Global Ecology and Biogeography. 2003;12:361–371. 10.1046/j.1466-822X.2003.00042.x

[5] Barrett RDH, Schluter D. Adaptation from standing genetic variation. Trends in Ecology & Evolution. 2008;23:38–44. 10.1016/j.tree.2007.09.008 18006185

[6] Neale DB, Kremer A. Forest tree genomics: growing resources and applications. Nat Rev Genet. 2011;12:111–122. 10.1038/nrg2931 21245829

[7] Murray BG. Nuclear DNA Amounts in Gymnosperms. Ann Bot. 1998;82:3–15.

[8] Neale DB, Savolainen O. Association genetics of complex traits in conifers. Trends in Plant Science. 2004;9:325–330. 10.1016/j.tplants.2004.05.006 15231277

[9] Eveno E, Collada C, Guevara MA, Léger V, Soto A, Díaz L, et al “Contrasting Patterns of Selection at Pinus pinaster Ait. Drought Stress Candidate Genes as Revealed by Genetic Differentiation Analyses.” Mol Biol Evol. 2008;25:417–437. 10.1093/molbev/msm272 18065486

[10] Müller T, Freund F, Wildhagen H, Schmid KJ. Targeted re-sequencing of five Douglas-fir provenances reveals population structure and putative target genes of positive selection. Tree Genetics & Genomes. 2015;11:1–17. 10.1007/s11295-014-0816-z

[11] Sork VL, Aitken SN, Dyer RJ, Eckert AJ, Legendre P, Neale DB. Putting the landscape into the genomics of trees: approaches for understanding local adaptation and population responses to changing climate. Tree Genetics & Genomes. 2013;9:901–911. 10.1007/s11295-013-0596-x

[12] Born N, Behringer D, Liepelt S, Beyer S, Schwerdtfeger M, Ziegenhagen B, et al Monitoring Plant Drought Stress Response Using Terahertz Time-Domain Spectroscopy. Plant Physiol. 2014;164:1571–1577. 10.1104/pp.113.233601 24501000 PMC3982723

[13] Konnert M, Bergmann F. The geographical distribution of genetic variation of silver fir (Abies alba, Pinaceae) in relation to its migration history. Pl Syst Evol. 1995;196:19–30. 10.1007/BF00985333

[14] Macias M, Andreu L, Bosch O, Camarero JJ, Gutiérrez E. Increasing Aridity is Enhancing Silver Fir Abies Alba Mill.) Water Stress in its South-Western Distribution Limit. Climatic Change. 2006;79:289–313. 10.1007/s10584-006-9071-0

[15] Lebourgeois F. Climatic signal in annual growth variation of silver fir (Abies alba Mill.) and spruce (Picea abies Karst.) from the French Permanent Plot Network (RENECOFOR). Ann For Sci. 2007;64:333–343. 10.1051/forest:20070101

[16] Toromani E, Sanxhaku M, Pasho E. Growth responses to climate and drought in silver fir (Abies alba) along an altitudinal gradient in southern Kosovo. Can J For Res. 2011;41:1795–1807. 10.1139/x11-096

[17] Peguero-Pina JJ, Camarero JJ, Abadía A, Martín E, González-Cascón R, Morales F, et al Physiological performance of silver-fir (Abies alba Mill.) populations under contrasting climates near the south-western distribution limit of the species. Flora—Morphology, Distribution, Functional Ecology of Plants. 2007;202:226–236. 10.1016/j.flora.2006.06.004

[18] Piovani P, Leonardi S, Magnani F, Menozzi P. Variability of stomatal conductance in a small and isolated population of silver fir (Abies alba Mill.). Tree Physiol. 2011;31:500–507. 10.1093/treephys/tpr029 21636691

[19] Linares JC, Camarero JJ. Silver Fir Defoliation Likelihood Is Related to Negative Growth Trends and High Warming Sensitivity at Their Southernmost Distribution Limit. ISRN Forestry. 2012;2012:e437690 10.5402/2012/437690

[20] Cailleret M, Nourtier M, Amm A, Durand-Gillmann M, Davi H. Drought-induced decline and mortality of silver fir differ among three sites in Southern France. Annals of Forest Science. 2013;1–15. 10.1007/s13595-013-0265-0

[21] Desprez-Loustau M-L, Marçais B, Nageleisen L-M, Piou D, Vannini A. Interactive effects of drought and pathogens in forest trees. Annals of Forest Science. 2006;63:597–612. 10.1051/forest:2006040

[22] Durand-Gillmann M, Cailleret M, Boivin T, Nageleisen L-M, Davi H. Individual vulnerability factors of Silver fir (Abies alba Mill.) to parasitism by two contrasting biotic agents: mistletoe (Viscum album L. ssp. abietis) and bark beetles (Coleoptera: Curculionidae: Scolytinae) during a decline process. Annals of Forest Science. 2012;1–15. 10.1007/s13595-012-0251-y

[23] Nourtier M, Chanzy A, Cailleret M, Yingge X, Huc R, Davi H. Transpiration of silver Fir (Abies alba mill.) during and after drought in relation to soil properties in a Mediterranean mountain area. Annals of Forest Science. 2012;1–13. 10.1007/s13595-012-0229-9

[24] Hansen OK, Vendramin GG, Sebastiani F, Edwards KJ. Development of microsatellite markers in Abies nordmanniana (Stev.) Spach and cross-species amplification in the Abies genus. Molecular Ecology Notes. 2005;5:784–787. 10.1111/j.1471-8286.2005.01062.x

[25] Kahl G, Molina C, Rotter B, Jüngling R, Frank A, Krezdorn N, et al Reduced representation sequencing of plant stress transcriptomes. J Plant Biochem Biotechnol. 2012;21:119–127. 10.1007/s13562-012-0129-y

[26] Yakovlev I, Fossdal CG, Skrøppa T, Olsen JE, Jahren AH, Johnsen Ø. An adaptive epigenetic memory in conifers with important implications for seed production. Seed Science Research. 2012;22:63–76. 10.1017/S0960258511000535

[27] Lenz TL, Eizaguirre C, Rotter B, Kalbe M, Milinski M. Exploring local immunological adaptation of two stickleback ecotypes by experimental infection and transcriptome-wide digital gene expression analysis. Molecular Ecology. 2013;22:774–786. 10.1111/j.1365-294X.2012.05756.x 22971109 PMC3579235

[28] Wang L, Feng Z, Wang X, Wang X, Zhang X. DEGseq: an R package for identifying differentially expressed genes from RNA-seq data. Bioinformatics. 2010;26:136–138. 10.1093/bioinformatics/btp612 19855105

[29] Rivals I, Personnaz L, Taing L, Potier M-C. Enrichment or depletion of a GO category within a class of genes: which test? Bioinformatics. 2007;23:401–407. 10.1093/bioinformatics/btl633 17182697

[30] Anders S, Huber W. Differential expression analysis for sequence count data. Genome Biology. 2010;11:R106 10.1186/gb-2010-11-10-r106 20979621 PMC3218662

[31] Tarazona S, García-Alcalde F, Dopazo J, Ferrer A, Conesa A. Differential expression in RNA-seq: A matter of depth. Genome Res. 2011;21:2213–2223. 10.1101/gr.124321.111 21903743 PMC3227109

[32] Bustin SA, Benes V, Garson JA, Hellemans J, Huggett J, Kubista M, et al The MIQE Guidelines: Minimum Information for Publication of Quantitative Real-Time PCR Experiments. Clinical Chemistry. 2009;55:611–622. 10.1373/clinchem.2008.112797 19246619

[33] Vandesompele J, Preter KD, Pattyn F, Poppe B, Roy NV, Paepe AD, et al Accurate normalization of real-time quantitative RT-PCR data by geometric averaging of multiple internal control genes. Genome Biology. 2002;3:research0034 10.1186/gb-2002-3-7-research0034 12184808 PMC126239

[34] Andersen CL, Jensen JL, Ørntoft TF. Normalization of Real-Time Quantitative Reverse Transcription-PCR Data: A Model-Based Variance Estimation Approach to Identify Genes Suited for Normalization, Applied to Bladder and Colon Cancer Data Sets. Cancer Res. 2004;64:5245–5250. 10.1158/0008-5472.CAN-04-0496 15289330

[35] Kubista M, Rusnakova V, Sec D, Sjögreen B, Tichopad A. GenEx: Data Analysis Software Quantitative Real-time PCR in Applied (ed Filion M) Microbiology. Horizon Scientific Press; 2012 pp. 63–84.

[36] Pfaffl MW, Horgan GW, Dempfle L. Relative expression software tool (REST©) for group-wise comparison and statistical analysis of relative expression results in real-time PCR. Nucl Acids Res. 2002;30:e36–e36. 10.1093/nar/30.9.e36 11972351 PMC113859

[37] Liu W, Saint DA. A New Quantitative Method of Real Time Reverse Transcription Polymerase Chain Reaction Assay Based on Simulation of Polymerase Chain Reaction Kinetics. Analytical Biochemistry. 2002;302:52–59. 10.1006/abio.2001.5530 11846375

[38] Pfaffl MW. 3.2. Markers of a Successful Real-Time RT-PCR Assay A-Z of quantitative PCR (ed SA Bustin). La Jolla, CA: International University Line; 2004 pp. 90–106.

[39] Tognetti VB, Aken OV, Morreel K, Vandenbroucke K, Cotte B van de, Clercq ID, et al Perturbation of Indole-3-Butyric Acid Homeostasis by the UDP-Glucosyltransferase UGT74E2 Modulates Arabidopsis Architecture and Water Stress Tolerance. Plant Cell. 2010;22:2660–2679. 10.1105/tpc.109.071316 20798329 PMC2947170

[40] Brunner AM, Yakovlev IA, Strauss SH. Validating internal controls for quantitative plant gene expression studies. BMC Plant Biology. 2004;4:14 10.1186/1471-2229-4-14 15317655 PMC515301

[41] Nicot N, Hausman J-F, Hoffmann L, Evers D. Housekeeping gene selection for real-time RT-PCR normalization in potato during biotic and abiotic stress. J Exp Bot. 2005;56:2907–2914. 10.1093/jxb/eri285 16188960

[42] Palovaara J, Hakman I. Conifer WOX-related homeodomain transcription factors, developmental consideration and expression dynamic of WOX2 during Picea abies somatic embryogenesis. Plant Mol Biol. 2008;66:533–549. 10.1007/s11103-008-9289-5 18209956

[43] Gonçalves S, Cairney J, Maroco J, Oliveira MM, Miguel C. Evaluation of control transcripts in real-time RT-PCR expression analysis during maritime pine embryogenesis. Planta. 2005;222:556–563. 10.1007/s00425-005-1562-0 16034587

[44] Tenea GN, Bota AP, Raposo FC, Maquet A. Reference genes for gene expression studies in wheat flag leaves grown under different farming conditions. BMC Research Notes. 2011;4:373 10.1186/1756-0500-4-373 21951810 PMC3193821

[45] Furuichi T, Cunningham KW, Muto S. A Putative Two Pore Channel AtTPC1 Mediates Ca2+ Flux in Arabidopsis Leaf Cells. Plant Cell Physiol. 2001;42:900–905. 10.1093/pcp/pce145 11577183

[46] Kurusu T, Yagala T, Miyao A, Hirochika H, Kuchitsu K. Identification of a putative voltage-gated Ca2+ channel as a key regulator of elicitor-induced hypersensitive cell death and mitogen-activated protein kinase activation in rice. The Plant Journal. 2005;42:798–809. 10.1111/j.1365-313X.2005.02415.x 15941394

[47] Wang Y-J, Yu J-N, Chen T, Zhang Z-G, Hao Y-J, Zhang J-S, et al Functional analysis of a putative Ca2+ channel gene TaTPC1 from wheat. J Exp Bot. 2005;56:3051–3060. 10.1093/jxb/eri302 16275671

[48] Konczal M, Koteja P, Stuglik MT, Radwan J, Babik W. Accuracy of allele frequency estimation using pooled RNA-Seq. Mol Ecol Resour. 2014;14:381–392. 10.1111/1755-0998.12186 24119300

[49] Pauletto M, Milan M, Moreira R, Novoa B, Figueras A, Babbucci M, et al Deep transcriptome sequencing of Pecten maximus hemocytes: A genomic resource for bivalve immunology. Fish & Shellfish Immunology. 2014;37: 154–165. 10.1016/j.fsi.2014.01.017 24486903

[50] Pinosio S, González-Martínez SC, Bagnoli F, Cattonaro F, Grivet D, Marroni F, et al. First insights into the transcriptome and development of new genomic tools of a widespread circum-Mediterranean tree species, Pinus halepensis Mill. Molecular Ecology Resources. 2014;n/a–n/a. 10.1111/1755-0998.12232 24450970

[51] Zhao X, Yu H, Kong L, Liu S, Li Q. Comparative Transcriptome Analysis of Two Oysters, Crassostrea gigas and Crassostrea hongkongensis Provides Insights into Adaptation to Hypo-Osmotic Conditions. PLoS ONE. 2014;9: e111915 10.1371/journal.pone.0111915 25369077 PMC4219811

[52] Zheng X, Moriyama EN. Comparative studies of differential gene calling using RNA-Seq data. BMC Bioinformatics. 2013;14:S7 10.1186/1471-2105-14-S13-S7 24267181 PMC3891352

[53] Guo Y, Li C-I, Ye F, Shyr Y. Evaluation of read count based RNAseq analysis methods. BMC Genomics. 2013;14:S2 10.1186/1471-2164-14-S8-S2 24564449 PMC4092879

[54] Dubos C, Plomion C. Identification of water-deficit responsive genes in maritime pine (Pinus pinaster Ait.) roots. Plant Mol Biol. 2003;51:249–262. 10.1023/A:1021168811590 12602883

[55] Fossdal CG, Nagy NE, Johnsen Ø, Dalen LS. Local and systemic stress responses in Norway spruce: Similarities in gene expression between a compatible pathogen interaction and drought stress. Physiological and Molecular Plant Pathology. 2007;70:161–173. 10.1016/j.pmpp.2007.09.002

[56] Velasco-Conde T, Yakovlev I, Majada JP, Aranda I, Johnsen Ø. Dehydrins in maritime pine (Pinus pinaster) and their expression related to drought stress response. Tree Genetics & Genomes. 2012;8:957–973. 10.1007/s11295-012-0476-9

[57] González-Martínez SC, Ersoz E, Brown GR, Wheeler NC, Neale DB. DNA Sequence Variation and Selection of Tag Single-Nucleotide Polymorphisms at Candidate Genes for Drought-Stress Response in Pinus taeda L. Genetics. 2006;172:1915–1926. 10.1534/genetics.105.047126 16387885 PMC1456261

[58] Eveno E, Collada C, Guevara MA, Léger V, Soto A, Díaz L, et al “Contrasting Patterns of Selection at Pinus pinaster Ait. Drought Stress Candidate Genes as Revealed by Genetic Differentiation Analyses.” Mol Biol Evol. 2008;25:417–437. 10.1093/molbev/msm272 18065486

[59] Perdiguero P, Collada C, Barbero M del C, García Casado G, Cervera MT, Soto Á. Identification of water stress genes in Pinus pinaster Ait. by controlled progressive stress and suppression-subtractive hybridization. Plant Physiology and Biochemistry. 2012;50:44–53. 10.1016/j.plaphy.2011.09.022 22099518

[60] Todd J, Post-Beittenmiller D, Jaworski JG. KCS1encodes a fatty acid elongase 3-ketoacyl-CoA synthase affecting wax biosynthesis inArabidopsis thaliana. The Plant Journal. 1999;17:119–130. 10.1046/j.1365-313X.1999.00352.x59 10074711

[61] Hamanishi ET, Campbell MM. Genome-wide responses to drought in forest trees. Forestry. 2011; 10.1093/forestry/cpr012

[62] Maurel C, Verdoucq L, Luu D-T, Santoni V. Plant Aquaporins: Membrane Channels with Multiple Integrated Functions. Annual Review of Plant Biology. 2008;59:595–624. 10.1146/annurev.arplant.59.032607.092734 18444909

[63] Fulton DC, Stettler M, Mettler T, Vaughan CK, Li J, Francisco P, et al β-AMYLASE4, a Noncatalytic Protein Required for Starch Breakdown, Acts Upstream of Three Active β-Amylases in Arabidopsis Chloroplasts. Plant Cell. 2008;20:1040–1058. 10.1105/tpc.107.056507 18390594 PMC2390740

[64] Cosgrove DJ. Growth of the plant cell wall. Nature Reviews Molecular Cell Biology. 2005;6:850–861. 10.1038/nrm1746 16261190

[65] Singh A, Giri J, Kapoor S, Tyagi AK, Pandey GK. Protein phosphatase complement in rice: genome-wide identification and transcriptional analysis under abiotic stress conditions and reproductive development. BMC Genomics. 2010;11:435 10.1186/1471-2164-11-435 20637108 PMC3091634

[66] Bu Q, Li H, Zhao Q, Jiang H, Zhai Q, Zhang J, et al The Arabidopsis RING Finger E3 Ligase RHA2a Is a Novel Positive Regulator of Abscisic Acid Signaling during Seed Germination and Early Seedling Development. Plant Physiol. 2009;150:463–481. 10.1104/pp.109.135269 19286935 PMC2675735

[67] Goyal K, Walton LJ, Tunnacliffe A. LEA proteins prevent protein aggregation due to water stress. Biochemical Journal. 2005;388:151 10.1042/BJ20041931 15631617 PMC1186703

[68] Yang Y, He M, Zhu Z, Li S, Xu Y, Zhang C, et al Identification of the dehydrin gene family from grapevine species and analysis of their responsiveness to various forms of abiotic and biotic stress. BMC Plant Biology. 2012;12:140 10.1186/1471-2229-12-140 22882870 PMC3460772

[69] Shinozaki K, Yamaguchi-Shinozaki K. Gene networks involved in drought stress response and tolerance. J Exp Bot. 2007;58:221–227. 10.1093/jxb/erl164 17075077

[70] Taji T, Ohsumi C, Iuchi S, Seki M, Kasuga M, Kobayashi M, et al Important roles of drought- and cold-inducible genes for galactinol synthase in stress tolerance in Arabidopsis thaliana. Plant J. 2002;29:417–426. 11846875 10.1046/j.0960-7412.2001.01227.x

[71] Malehorn DE, Borgmeyer JR, Smith CE, Shah DM. Characterization and Expression of an Antifungal Zeamatin-like Protein (Zlp) Gene from Zea mays. Plant Physiol. 1994;106:1471–1481. 10.1104/pp.106.4.1471 7846159 PMC159687

[72] Wu S, Kriz AL, Widholm JM. Nucleotide Sequence of a Maize cDNA for a Class II, Acidic [beta]-1,3-Glucanase. Plant Physiol. 1994;106:1709–1710. 10.1104/pp.106.4.1709 7846180 PMC159727

[73] Witmer X, Nonogaki H, Beers EP, Bradford KJ, Welbaum GE. Characterization of chitinase activity and gene expression in muskmelon seeds. Seed Science Research. 2003;13:167–178. 10.1079/SSR2003134

[74] Bonardi V, Tang S, Stallmann A, Roberts M, Cherkis K, Dangl JL. Expanded functions for a family of plant intracellular immune receptors beyond specific recognition of pathogen effectors. PNAS. 2011;108:16463–16468. 10.1073/pnas.1113726108 21911370 PMC3182704

[75] Sheehan D, Meade G, Foley VM, Dowd CA. Structure, function and evolution of glutathione transferases: implications for classification of non-mammalian members of an ancient enzyme superfamily. Biochem J. 2001;360:1–16. 11695986 10.1042/0264-6021:3600001 PMC1222196

[76] Siqueira M, Gomes L. Functional Diversity of Early Responsive to Dehydration (ERD) Genes in Soybean In: Board J, editor. A Comprehensive Survey of International Soybean Research—Genetics, Physiology, Agronomy and Nitrogen Relationships. InTech; 2013 Available: http://www.intechopen.com/books/a-comprehensive-survey-of-international-soybean-research-genetics-physiology-agronomy-and-nitrogen-relationships/functional-diversity-of-early-responsive-to-dehydration-erd-genes-in-soybean 10.1016/j.yrtph.2010.06.017

[77] Ralph SG, Chun H, Kolosova N, Cooper D, Oddy C, Ritland CE, et al A conifer genomics resource of 200,000 spruce (Picea spp.) ESTs and 6,464 high-quality, sequence-finished full-length cDNAs for Sitka spruce (Picea sitchensis). BMC Genomics. 2008;9:484 10.1186/1471-2164-9-484 18854048 PMC2579922

