# Supplementary material for: Differential Gene Expression Reveals Candidate Genes for Drought Stress Response in Abies alba (Pinaceae)
Source: PLoS One. 2015 Apr 29;10(4):e0124564. doi: 10.1371/journal.pone.0124564 (PMC4414588; doi:10.1371/journal.pone.0124564)
Supplement: S1 File — (DOCX) [file pone.0124564.s001.docx]

Supporting information File S1

Differential gene expression reveals candidate genes for drought stress response in *Abies alba* (Pinaceae)

David Behringer^1¶*^, Heike Zimmermann^1,#a¶^, Birgit Ziegenhagen^1^, Sascha Liepelt^1^

^1^Conservation Biology Group, Philipps-University of Marburg, Marburg, Germany

^#a^Current address: Alfred-Wegener-Institute for Polar and Marine Research, Research Unit Potsdam, Department of Periglacial Research, Potsdam, Germany

* Corresponding author

E-mail: david.behringer@biologie.uni-marburg.de (DB)

^¶^These authors contributed equally to this work.

**Table S1. Temperature and cycle program for a qPCR run on the Light Cycler 480 II.** The annealing temperature (*) varied depending on the primer pair.

| Program Name | Pre-incubation | | | |
| --- | --- | --- | --- | --- |
| Cycles | 1 | Analysis mode | None | |
| Target | Aquisition Mode | hold (hh:mm:ss) | Ramp Rate (°C/s) | Aquisitions (per °C) |
| 95 | None | 00:03:00 | 4.40 |  |
|  |  |  |  |  |
| Program Name | Amplification | | | |
| Cycles | 55 | Analysis mode | Quantification | |
| Target | Aquisition Mode | hold (hh:mm:ss) | Ramp Rate (°C/s) | Aquisitions (per °C) |
| 95 | None | 00:00:03 | 4.40 |  |
| 59* | None | 00:00:25 | 2.20 |  |
| 72 | Single | 00:00:15 | 4.40 |  |
|  |  |  |  |  |
| Program Name | Melting curve | | | |
| Cycles | 1 | Analysis mode | Melting Curves | |
| Target | Aquisition Mode | hold (hh:mm:ss) | Ramp Rate (°C/s) | Aquisitions (per °C) |
| 95 | None | 00:00:05 | 4.40 |  |
| 65 | None | 00:01:00 | 2.20 |  |
| 97 | Continuous |  | 0.11 | 5 |
|  |  |  |  |  |
| Program Name | Cooling | | | |
| Cycles | 1 | Analysis mode | None | |

**Table S2. Genes which were selected and tested for validation by qPCR and reference genes identified in the MACE dataset with primer name (combination of transcript name and possible isoform, see Table E-H), forward and reverse primer sequence, estimated annealing temperatures (Tm) and expected product size.**

| **Primer name** | **Tm [°C]** | **Primer forward (5' → 3')** | **Tm [°C]** | **Primer reverse (5' → 3')** | **Size [bp]** |
| --- | --- | --- | --- | --- | --- |
| At4g33300 | 61 | AGACAGCAAGGAAGAGATCAAGA | 60 | TGATAACTGTGCAGAGGAAAGC | 75 |
| BAM1.a | 58 | ACGTTGAGGCATGGAGAAGA | 58 | TGAGATTTCCAGCGGCATTG | 67 |
| BAM1.b* | 60 | TGGAGGTTGTTCGTCTCATTTG | 58 | CAGCAGCTTCTTGGACCATT | 130 |
| Cht8 | 58 | ACTCACTCTTGTATGCTGCG | 60 | ACAACCCTAAGCCTGGAGAC | 133 |
| CIPK17 | 56 | AGCTGTGCACAAAGTTAGCA | 60 | CATTCCTTATGTGGCCCTCC | 70 |
| CXE15/2 | 60 | TGCATTTCTCCCTCTGATCACT | 60 | GCAACGTGTGGGAGAAGATC | 146 |
| dhn2 | 58 | CACACCGGTTTGATGGGAAA | 62 | CCTCCCTCTTCATCGCTCG | 74 |
| ERD10* | 60 | GCCAGAGTCAGCCTGAGAAT | 58 | ATTTTATCGAGAAGCCCCGC | 76 |
| GLU.a1.4 | 59 | GAAGCCAGGAACGAGCATC | 60 | CCGCCTTCTTGTTCTCGTTG | 62 |
| GLU.a10.2 | 60 | CCCATGTGGCCAAGTCCTTA | 60 | ACCGTAAGTGGAAGGAAGATCT | 71 |
| GLU.a10.3.6 | 60 | ACGAGAACAAGAAGACGGGG | 58 | TGCAGGAGTTTGATCAGGGT | 71 |
| GLU.a15 | 58 | ACCCTGATCAAACTCCTGCA | 56 | TGAGGTGAAGTCAAACGCAA | 69 |
| GOLS2.a | 58 | ATCAACTCTGCAGCAAGTGG | 60 | GAGGAGCAGGCATCAAGTTG | 77 |
| GOLS2.b | 59 | TCCAATTTCGGAGGCGGAC | 58 | CGGAGCAGGCATCCAGTT | 64 |
| GPPS3 | 58 | TCCTTCAGTCTTGGTTGCGA | 60 | CTCTTTCCGCCGCTGAATTG | 65 |
| GSTU19 | 60 | GGGTATATGGGCCGTTGGAT | 58 | TTTGTCAAAGTGGATGCTAACC | 100 |
| GTE8^R^ | 60 | GATGTGACGGACTGGATTCC | 58 | TATGGGCTGTGCATTCTGTG | 75 |
| INR1 | 58 | TAGCCTGCCTACCAAACCTT | 60 | ATGAAGGCGACGAGTAGGTC | 148 |
| KCS11.a | 60 | GCCCTACGGACTATCAATCC | 60 | CCCTAGGAACATCAACAGGG | 82 |
| KCS11.b | 58 | ACTGGGTTCGGGATTCAAGT | 58 | ATCCGGTCATCCCAAACAGT | 90 |
| LEA protein | 60 | GAAAGTGCCAGAGGAAAGCC | 60 | CATCACCATGCTTTCCCTCG | 77 |
| LEA14-A | 60 | CTTGGAGAGAGGAAGGGTGA | 58 | ACGTTGGAAGGGATTGGGAT | 95 |
| LTI6B.a | 60 | GTCTGCTGTTGACTGTGCTC | 58 | ACATGTGACCAAACTCCAAACA | 111 |
| LTI6B.b | 58 | TGGCCATAATTCTGCCTCCT | 58 | AGAACTCAGCATTGCAACCG | 60 |
| MGL | 58 | TCCAGTGTCATTGCTAGGCT | 57 | ACAAAACATAAAGGTGCCACAA | 66 |
| Os01g0656200 | 60 | ATGGCCCGTATAGAGGATGC | 58 | ATCTGGCTCGGGAATAACGT | 129 |
| Pat3 | 58 | GCAGAACAGCTTGTCATGGT | 56 | ACGACGCTTTCTTCAAACCA | 66 |
| PPO_A1 | 60 | AGGGCGAGAAAAGAGTGAGG | 60 | ACAGTAACACTCTCCGCCTC | 88 |
| PUP1.a1.12 | 56 | TGGAACATCGACCGACAATT | 60 | GTGCAAATCTCGTCCGATCC | 120 |
| PUP1.a10.12.7 | 59 | AGGCCAGACTCAAACTACTCA | 58 | ATTGTCGGTCGATGTTCCAG | 140 |
| PUP1.a10.13.9* | 59 | AGGCCAGACTCAAACTACTCA | 60 | TCCGCCATCCCCTTATTCTC | 63 |
| PUP1.a11.13.8 | 57 | TTTGTTTTGATTTTCGTGGCGA | 61 | GAAACTCTGCTGATCATCCCC | 63 |
| PUP1.a11.5 | 59 | CCTGGAGGCAAAAGCAAGG | 60 | CTGTCGGTCGATGTTTCAGG | 60 |
| PUP1.a2.7 | 60 | ATAAGGGGATGGCGGAGAAG | 60 | TTGTCGGTCGATGTTCCAGG | 80 |
| PUP3 | 58 | GACTTCATGCATGCTCGTGA | 60 | TTGAAACATCCCCTCCCGAG | 60 |
| PUP4 | 58 | TTCCCGAAGACCCCAAATCA | 59 | TCATCCTTACACACAGCCTCT | 61 |
| RCI2A | 58 | TTCCTGTGTTGGCTCTCTGA | 58 | TGCCCTCCCTCATTGTGATT | 69 |
| RHA2A.b.2.5.6 | 58 | CCAGATTCAAGTTGCAGCCA | 58 | AAGATGAGGTGGTTGTGGCT | 124 |
| RHA2A.a1 | 60 | AAACCCAGTCCAGTCGGTAG | 58 | ACAGAGATGCGTGGGAAACT | 90 |
| RHA2A.a.1.2 | 60 | ATTAGGAGTCTGCCCTGTGG | 60 | AGAGGGCATGTCATATTCTCGT | 86 |
| RHA2A.b1.2.6 | 58 | GCCACAACCACCTCATCTTT | 61 | CACCACTTTGCAGAACTACTACA | 61 |
| RHA2A.b.2.4 | 60 | TCACTCCCTGCTAGATTTTCGA | 59 | GCAATGGAACGGTTATGTCGA | 138 |
| STP13 | 60 | GAATCACCTTCCCCAATGCC | 58 | AGCGTGAGACTGCAACTACT | 61 |
| TIF2A^R^ | 58 | CCATGAAGGCAATTACGGGA | 56 | ACACAAGTGACAAAGCCAGT | 61 |
| TIP1-1 | 60 | GTGCGATAAAGTGAGGGCTG | 60 | CGAGACGACAAAGCGATGAG | 115 |
| TPCC^R^ | 58 | GTTGTGACATGCCAGCCTAA | 57 | TGCCGACATGTCACACCAT | 131 |
| UGT74E2 | 58 | AGAAATTAGCGAGGGAGGCA | 58 | AATGCCTTGGCAGCAATCTC | 88 |
| VTE4 | 58 | ACTGCTCAGAAACCTGCATG | 58 | AGCCTCCTCCATCACAGAAA | 97 |
| XTH6 | 57 | CAGCGTTCGTTGTGCTTCT | 60 | TCACCCACCTGTAGAGCTTG | 77 |
| XTH7* | 60 | CCCTGTCACACCTCCAGAAT | 59 | GGTTCAACGTTCATCACACCA | 150 |

^R^Reference gene, *Specific amplification during RT-qPCR.

GLU=Glucan-endo-1,3-beta-glucosidase, acidic isoform; Pat3=Patatin-like protein 3; PPO_A1=Polyphenol oxidase A1; PUP=Putative uncharacterized protein.

**Table S3. Most enriched GO terms of the GO domain biological process on GO level 3.** Sorted by enrichment-*p*-value in ascending order, up- and down-regulated refer to transcripts.

| **GO term identifier** | **GO term description** | **Transcripts** | **DETs (*p*-value<1E-10)** | **Up-regulated** | **Down-regulated** | **Enrichment-*p*-value** | **GO-Level(s)** |
| --- | --- | --- | --- | --- | --- | --- | --- |
| GO:0044710 | single-organism metabolic process | 9320 | 514 | 195 | 319 | 1.18E-24 | 3 |
| GO:0044237 | cellular metabolic process | 21467 | 945 | 361 | 584 | 8.10E-16 | 3 |
| GO:0071704 | organic substance metabolic process | 21486 | 936 | 384 | 552 | 3.51E-14 | 3 |
| GO:0006950 | response to stress | 8218 | 405 | 250 | 155 | 3.33E-11 | 3 |
| GO:0044238 | primary metabolic process | 20072 | 849 | 355 | 494 | 4.20E-09 | 3 |
| GO:0071554 | cell wall organization or biogenesis | 638 | 50 | 22 | 28 | 4.66E-07 | 3 |
| GO:0009628 | response to abiotic stimulus | 5522 | 262 | 155 | 107 | 4.82E-06 | 3 |
| GO:0031640 | killing of cells of other organism | 15 | 6 | 2 | 4 | 8.82E-06 | 3 |
| GO:0042221 | response to chemical stimulus | 6466 | 297 | 164 | 133 | 1.34E-05 | 3 |
| GO:0032259 | methylation | 388 | 31 | 4 | 27 | 4.61E-05 | 3 |
| GO:0009058 | biosynthetic process | 13025 | 547 | 182 | 365 | 5.49E-05 | 3 |
| GO:0009607 | response to biotic stimulus | 2415 | 119 | 67 | 52 | 0.00058978 | 3 |
| GO:0042445 | hormone metabolic process | 287 | 22 | 16 | 6 | 0.00094645 | 3 |
| GO:0051707 | response to other organism | 2347 | 107 | 60 | 47 | 0.01098406 | 3 |
| GO:0009056 | catabolic process | 5001 | 210 | 96 | 114 | 0.01694164 | 3 |
| GO:0009719 | response to endogenous stimulus | 2965 | 128 | 72 | 56 | 0.02758434 | 3 |
| GO:0022611 | dormancy process | 72 | 6 | 3 | 3 | 0.04764202 | 3 |
| GO:0048585 | negative regulation of response to stimulus | 399 | 28 | 15 | 13 | 0.0008441 | 3,5 |
| GO:0043901 | negative regulation of multi-organism process | 10 | 2 | 2 | 0 | 0.04930459 | 3,5 |
| GO:0010073 | meristem maintenance | 166 | 12 | 5 | 7 | 0.01911781 | 3,6 |

DETs=differentially expressed transcripts.

**Table S4. Top 40 most enriched GO terms of the GO domain biological process on GO level 4.** Sorted by enrichment-*p*-value in ascending order, up- and down-regulated refer to transcripts.

| **GO term identifier** | **GO term description** | **Transcripts** | **DETs (*p*-value<1e-10)** | **Up-regulated** | **Down-regulated** | **Enrichment-*p*-value** |
| --- | --- | --- | --- | --- | --- | --- |
| GO:0055114 | oxidation-reduction process | 3029 | 259 | 94 | 165 | 2.87E-38 |
| GO:0015979 | photosynthesis | 1260 | 116 | 10 | 106 | 1.03E-19 |
| GO:0005975 | carbohydrate metabolic process | 3161 | 211 | 91 | 120 | 9.90E-18 |
| GO:1901700 | response to oxygen-containing compound | 3703 | 234 | 138 | 96 | 7.67E-17 |
| GO:0072593 | reactive oxygen species metabolic process | 319 | 43 | 13 | 30 | 2.00E-13 |
| GO:0009415 | response to water stimulus | 1011 | 87 | 65 | 22 | 2.03E-13 |
| GO:0010035 | response to inorganic substance | 2474 | 163 | 93 | 70 | 2.28E-13 |
| GO:0019748 | secondary metabolic process | 1417 | 109 | 42 | 67 | 2.87E-13 |
| GO:0006091 | generation of precursor metabolites and energy | 2061 | 133 | 26 | 107 | 1.68E-10 |
| GO:0044281 | small molecule metabolic process | 6065 | 307 | 119 | 188 | 1.12E-09 |
| GO:1901698 | response to nitrogen compound | 464 | 44 | 25 | 19 | 1.08E-08 |
| GO:0044262 | cellular carbohydrate metabolic process | 932 | 70 | 29 | 41 | 1.42E-08 |
| GO:0070887 | cellular response to chemical stimulus | 1784 | 112 | 50 | 62 | 2.15E-08 |
| GO:0009620 | response to fungus | 495 | 45 | 27 | 18 | 2.63E-08 |
| GO:0006952 | defense response | 2382 | 138 | 79 | 59 | 6.43E-08 |
| GO:0042180 | cellular ketone metabolic process | 779 | 59 | 22 | 37 | 1.44E-07 |
| GO:0044711 | single-organism biosynthetic process | 3247 | 171 | 57 | 114 | 8.80E-07 |
| GO:1901135 | carbohydrate derivative metabolic process | 1970 | 114 | 40 | 74 | 9.87E-07 |
| GO:0006970 | response to osmotic stress | 1229 | 79 | 43 | 36 | 1.08E-06 |
| GO:0009814 | defense response, incompatible interaction | 334 | 31 | 16 | 15 | 2.38E-06 |
| GO:0080167 | response to karrikin | 63 | 12 | 5 | 7 | 2.55E-06 |
| GO:0045087 | innate immune response | 586 | 45 | 27 | 18 | 2.92E-06 |
| GO:0071495 | cellular response to endogenous stimulus | 1106 | 71 | 34 | 37 | 3.91E-06 |
| GO:0016131 | brassinosteroid metabolic process | 56 | 11 | 10 | 1 | 4.86E-06 |
| GO:2001057 | reactive nitrogen species metabolic process | 70 | 12 | 5 | 7 | 8.04E-06 |
| GO:0044364 | disruption of cells of other organism | 15 | 6 | 2 | 4 | 8.82E-06 |
| GO:0006081 | cellular aldehyde metabolic process | 295 | 27 | 3 | 24 | 1.33E-05 |
| GO:1901576 | organic substance biosynthetic process | 12481 | 529 | 177 | 352 | 2.96E-05 |
| GO:0018904 | ether metabolic process | 45 | 9 | 3 | 6 | 3.04E-05 |
| GO:1901615 | organic hydroxy compound metabolic process | 714 | 48 | 21 | 27 | 4.52E-05 |
| GO:1901657 | glycosyl compound metabolic process | 919 | 58 | 14 | 44 | 4.66E-05 |
| GO:0044092 | negative regulation of molecular function | 126 | 15 | 4 | 11 | 5.88E-05 |
| GO:0045730 | respiratory burst | 29 | 7 | 4 | 3 | 6.56E-05 |
| GO:0019637 | organophosphate metabolic process | 2091 | 110 | 36 | 74 | 8.76E-05 |
| GO:0071941 | nitrogen cycle metabolic process | 96 | 12 | 5 | 7 | 0.00019761 |
| GO:0010191 | mucilage metabolic process | 10 | 4 | 2 | 2 | 0.00031107 |
| GO:0009617 | response to bacterium | 1235 | 69 | 39 | 30 | 0.00035946 |
| GO:0009414 | response to water deprivation | 921 | 54 | 33 | 21 | 0.00050419 |
| GO:0010243 | response to organic nitrogen | 332 | 25 | 15 | 10 | 0.0005757 |
| GO:0044249 | cellular biosynthetic process | 12380 | 511 | 171 | 340 | 0.00058483 |

DETs=differentially expressed transcripts.

**Table S5. Up-regulated transcripts in response to water stimulus, ordered alphabetically by transcript name.** Sense tags are the amount of different tags with match in sense orientation (5’3’) to a database entry. Possibly different protein isoforms are labeled with “a” and “b”, different database sequence hits with numbers. Fold change is derived from the DEGseq analysis.

| **Transcript name** | **Possible isoform** | **Blast score** | **Accession number** | **Source organism** | **Sense tags** | **TPM stressed** | **TPM control** | **Fold change** |
| --- | --- | --- | --- | --- | --- | --- | --- | --- |
| Aquaporin TIP1-1 | - | 87.4 | P25818 | *Arabidopsis thaliana* | 284 | 64.2821 | 1.1917 | 5.75332 |
| Beta-amylase 1, chloroplastic | a | 67.8 | Q9LIR6 | *A. thaliana* | 68 | 11.5998 | 0.1083 | 6.74292 |
| Beta-amylase 1, chloroplastic | b | 115 | Q9LIR6 | *A. thaliana* | 1614 | 577.895 | 57.4178 | 3.33124 |
| Dehydrin 2 (Fragment) | - | 85.1 | E1A556 | *Pinus pinaster* | 85 | 18.8497 | 0 | 8.5584 |
| Dehydrin ERD10 | - | 48.5 | P42759 | *A. thaliana* | 2372 | 1048.65 | 107.36 | 3.288 |
| GolS-1 or 2 | 1: 3 or 4 or 2: a5 | 53.9 or ? or 82.8 | Q947G8 or C7G304 | *Solanum lycopersicum* | 147 | 33.1883 | 1.0834 | 4.93704 |
| GolS2 | b2 | 84.7 | Q9FXB2 | *A. thaliana* | 57 | 15.4664 | 0 | 8.27299 |
| GolS-2 or 1 | 2: a1 or 1: 3 or 6 | 82.8 or 53.9 or 53.9 | C7G304 or Q947G8 | *S. lycopersicum* | 221 | 52.6824 | 2.1667 | 4.60375 |
| GST U19 | - | 47 | Q9ZRW8 | *A. thaliana* | 62 | 12.2442 | 0.1083 | 6.82092 |
| LEA protein | - | 49.7 | P21298 | *Raphanus sativus* | 7947 | 4564.19 | 510.91 | 3.15922 |
| LEA protein Lea14-A | - | 97.1 | P46518 | *Gossypium hirsutum* | 2512 | 1411.47 | 47.8843 | 4.8815 |
| MGL | - | 56.2 or 56.2 | Q9SGU9 | *A. thaliana* | 186 | 28.6772 | 3.5751 | 3.00385 |
| PUP1 | a1 or 10 or 9 | 115 or 115 or 115 | A9NLY4 | *Picea sitchensis* | 3674 | 1393.75 | 91.4352 | 3.93007 |
| PUP1 | a1 or 12 | 115 or 115 | A9NLY4 | *P. sitchensis* | 441 | 372.321 | 2.0584 | 7.49888 |
| PUP1 | a1 or 13 or 9 | 115 or 115 or 115 | A9NLY4 | *P. sitchensis* | 139 | 46.3991 | 4.6584 | 3.31619 |
| PUP1 | a2 or 6 or 8 | 115 or 115 or 115 | A9NLY4 | *P. sitchensis* | 601 | 425.164 | 25.2422 | 4.07411 |
| PUP1 | a2 or 7 | 115 or 115 | A9NLY4 | *P. sitchensis* | 391 | 392.137 | 28.2756 | 3.79373 |
| PUP1 | a2 or 8 | 115 or 115 | A9NLY4 | *P. sitchensis* | 63 | 27.3884 | 2.7084 | 3.33805 |
| PUP1 | a6 or 7 | 115 or 115 | A9NLY4 | *P. sitchensis* | 88 | 83.1318 | 1.5167 | 5.77639 |
| PUP1 | a10 or 12 or 7 | 115 or 115 or 115 | A9NLY4 | *P. sitchensis* | 370 | 176.736 | 4.5501 | 5.27955 |
| PUP1 | a10 or 13 or 9 | 115 or 115 or 115 | A9NLY4 | *P. sitchensis* | 276 | 196.713 | 17.3337 | 3.50444 |
| PUP1 | a10 or 2 or 9 | 115 or 115 or 115 | A9NLY4 | *P. sitchensis* | 465 | 397.132 | 32.8257 | 3.59672 |
| PUP1 | a10 or 9 | 115 or 115 | A9NLY4 | *P. sitchensis* | 97 | 73.3042 | 6.2835 | 3.54426 |
| PUP1 | a11 | 125 | A9NLY4 | *P. sitchensis* | 175 | 45.2714 | 3.7917 | 3.57768 |
| PUP1 | a11 or 13 or 8 | 125 or 115 or 115 | A9NLY4 | *P. sitchensis* | 564 | 281.617 | 22.6421 | 3.63665 |
| PUP1 | a11 or 5 | 125 or 125 | A9NLY4 | *P. sitchensis* | 185 | 60.4156 | 3.0334 | 4.31591 |
| PUP1 | b1 or 2 | 69.7 or 53.9 | A9NLY4 | *P. sitchensis* | 85 | 17.7219 | 1.5167 | 3.54653 |
| STP 13 | - | 82.4 | Q94AZ2 | *A. thaliana* | 474 | 119.381 | 2.6001 | 5.52086 |

TPM=tags per million; GolS=galactinol synthase, GST=glutathione S-transferase, MGL=methionine gamma-lyase, PUP=putative uncharacterized protein, STP=sugar transport protein; ?=no score available.

**Table S6. Up-regulated transcripts in response to osmotic stress, ordered alphabetically by transcript name.** Sense tags are the amount of different tags with match in sense orientation (5’3’) to a database entry. Possibly different protein isoforms are labeled with “a” and “b”, different database sequence hits with numbers. Fold change is derived from the DEGseq analysis.

| **Transcript name** | **Possible isoform** | **Blast score** | **Accession number** | **Source organism** | **Sense tags** | **TPM stressed** | **TPM control** | **Fold change** |
| --- | --- | --- | --- | --- | --- | --- | --- | --- |
| 3-ketoacyl-CoA synthase 11 | a | 66.6 | O48780 | *Arabidopsis thaliana* | 561 | 14.0836 | 143.2926 | 3.19762 |
| Aquaporin TIP1-1 | - | 87.4 | P25818 | *A. thaliana* | 284 | 1.1917 | 65.4738 | 5.75332 |
| CBL-interacting protein kinase 17 | - | 80.9 | Q75L42 | *Oryza sativa subsp. japonica* | 93 | 1.1917 | 16.9803 | 3.72779 |
| Dehydrin ERD10 | - | 48.5 | P42759 | *A. thaliana* | 2372 | 107.36 | 1156.01 | 3.288 |
| E3 ubiquitin-protein ligase RHA2A | a1 | 67.4 | Q9ZT50 | *A. thaliana* | 486 | 3.4667 | 146.2087 | 5.3637 |
| E3 ubiquitin-protein ligase RHA2A | a1 or 2 | 67.4 or 67.4 | Q9ZT50 | *A. thaliana* | 508 | 5.5251 | 183.8721 | 5.01254 |
| GolS2 | b2 | 84.7 | Q9FXB2 | *A. thaliana* | 57 | 0 | 15.4664 | 8.27299 |
| Glucan endo-1,3-beta-glucosidase, acidic isoform | 1 | 174 | P49237 | *Zea mays* | 124 | 0 | 30.1272 | 9.23492 |
| Glucan endo-1,3-beta-glucosidase, acidic isoform | 1 or 4 | 174 or 174 | P49237 | *Z. mays* | 115 | 0 | 30.2883 | 9.24262 |
| Glucan endo-1,3-beta-glucosidase, acidic isoform | 1 or 10 or 6 | 174 or 180 or 180 | P49237 | *Z. mays* | 92 | 0.1083 | 19.6024 | 7.49186 |
| Glucan endo-1,3-beta-glucosidase, acidic isoform | 10 or 2 | 180 or 157 | P49237 | *Z. mays* | 50 | 0.1083 | 9.4526 | 6.43098 |
| Glucan endo-1,3-beta-glucosidase, acidic isoform | 10 or 3 or 6 | 180 or 180 or 180 | P49237 | *Z. mays* | 58 | 0.4333 | 16.2219 | 5.18737 |
| Glucan endo-1,3-beta-glucosidase, acidic isoform | 15 | 139 | P49237 | *Z. mays* | 91 | 0.1083 | 22.8245 | 7.71254 |
| Glucan endo-1,3-beta-glucosidase, acidic isoform | 15 or 4 | 139 or 174 | P49237 | *Z. mays* | 144 | 0.2167 | 31.7939 | 7.18704 |
| GST U19 | - | 47 | Q9ZRW8 | *A. thaliana* | 62 | 0.1083 | 12.3525 | 6.82092 |
| Hydrophobic protein LTI6B | a | 99.8 | Q0DKW8 | *O. sativa subsp. japonica* | 94 | 0.325 | 16.4358 | 5.63144 |
| Hydrophobic protein LTI6B | b | 75.5 | Q0DKW8 | *O. sativa subsp. japonica* | 563 | 8.1252 | 194.5272 | 4.51987 |
| Hydrophobic protein RCI2A | - | 74.3 | Q9ZNQ7 | *A. thaliana* | 2087 | 51.676 | 892.177 | 4.02368 |
| STP 13 | - | 82.4 | Q94AZ2 | *A. thaliana* | 474 | 2.6001 | 121.9811 | 5.52086 |
| Zeamatin |  | 51.6 | P33679 | *Z. mays* | 130 | 1.8417 | 22.7858 | 3.50743 |

TPM=tags per million; GolS=galactinol synthase, GST=glutathione S-transferase, STP=sugar transport protein.

**Table S7. Down-regulated transcripts in response to water stimulus (RWS), water deprivation (RWD) and osmotic stress (all entries), ordered alphabetically by transcript name.** Sense tags are the amount of different tags with match in sense orientation (5’3’) to a database entry. Possibly different protein isoforms are labeled with “a” and “b”, different database sequence hits with numbers. Fold change is derived from the DEGseq analysis.

| **Transcript name** | **Possible isoform** | **Blast score** | **Accession number** | **Source organism** | **Sense tags** | **TPM stressed** | **TPM control** | **Fold change** |
| --- | --- | --- | --- | --- | --- | --- | --- | --- |
| 3-ketoacyl-CoA synthase 11 | b | 144 | O48780 | *Arabidopsis thaliana* | 279 | 0.9666 | 50.051 | -5.69434 |
| E3 ubiquitin-protein ligase RHA2A | b1 or 2 or 6 | 73.2 or 73.2 or 73.2 | Q9ZT50 | *A. thaliana* | 422 | 1.2889 | 71.8264 | -5.8003 |
| E3 ubiquitin-protein ligase RHA2A | b2 or 5 or 6 | 73.2 or 73.2 or 73.2 | Q9ZT50 | *A. thaliana* | 119 | 0.3222 | 20.9088 | -6.02001 |
| E3 ubiquitin-protein ligase RHA2A | b2 or 4 | 73.2 or 73.2 | Q9ZT50 | *A. thaliana* | 58 | 0.1611 | 8.0168 | -5.637 |
| Tubulin beta-8 chain | - | 144 | P29516 | *A. thaliana* | 117 | 1.6111 | 13.2169 | -3.03627 |
| UGT 74E2 (RWS/RWD) | - | 149 | Q9SYK9 | *A. thaliana* | 550 | 8.6998 | 77.6766 | -3.15843 |
| XTH-6 (RWS/RWD) | - | 203 | Q8LF99 | *A. thaliana* | 695 | 6.4443 | 122.961 | -4.25403 |

TPM=tags per million; XTH=probable xyloglucan endotransglucosylase/hydrolase protein, UGT=UDP-glycosyltransferase.

**Table S8. Transcripts with the ten highest and ten lowest fold changes; sorted by fold change, derived from the DEGseq analysis, in ascending and descending order, respectively.** Sense tags is the amount of different tags with match in sense orientation (5’3’) to a database entry; TPM=tags per million. Possibly different protein isoforms are labeled with “a” and “b”, different database sequence hits with numbers.

| **Transcript name** | **Possible isoform** | **Blast score** | **Accession number** | **Source organism** | **Sense tags** | **TPM stressed** | **TPM control** | **Fold change** |
| --- | --- | --- | --- | --- | --- | --- | --- | --- |
| Polyphenol oxidase A1 | - | 50.4 | Q06215 | *Vicia faba* | 368 | 78.1374 | 0 | 10.6099 |
| Probable protein phosphatase 2C 8 | - | 132 | Q5SN75 | *Oryza sativa subsp. japonica* | 830 | 243.918 | 0.325 | 9.55174 |
| Glucan endo-1,3-beta-glucosidase, acidic isoform | 1 or 4 | 174 or 174 | P49237 | *Zea mays* | 115 | 30.2883 | 0 | 9.24262 |
| Glucan endo-1,3-beta-glucosidase, acidic isoform | 1 | 174 | P49237 | *Z. mays* | 124 | 30.1272 | 0 | 9.23492 |
| PDR protein At4g33300 | - | 49.3 | Q9SZA7 | *Arabidopsis thaliana* | 109 | 19.8163 | 0 | 8.63054 |
| Dehydrin 2 (Fragment) | - | 85.1 | E1A556 | *Pinus pinaster* | 85 | 18.8497 | 0 | 8.5584 |
| PUP2 | - | 159 | A9NPH4 | *Picea sitchensis* | 731 | 196.552 | 0.5417 | 8.5032 |
| Probable carboxylesterase 15 or 2 | - | 80.9 or 72.4 | Q9FG13 or Q9SX78 | *A. thaliana* | 93 | 17.0775 | 0 | 8.41595 |
| Chitinase 8 | - | 62.8 | Q7XCK6 | *O. sativa subsp. japonica* | 194 | 35.9271 | 0.1083 | 8.3739 |
| GolS2 | b2 | 84.7 | Q9FXB2 | *A. thaliana* | 57 | 15.4664 | 0 | 8.27299 |
| PUP3 | - | 130 | F6HZZ7 | *Vitis vinifera* | 165 | 0 | 64.0263 | -10.3225 |
| Inducible nitrate reductase [NADH] 1 | - | 136 | P54233 | *Glycine max* | 370 | 0.1611 | 83.2017 | -9.01251 |
| Tocopherol O-methyltransferase | - | 134 | Q9ZSK1 | *A. thaliana* | 402 | 0.1611 | 68.0347 | -8.72217 |
| Patatin-like protein 3 | - | 58.2 | B6TPQ5 | *Z. mays* | 108 | 0 | 13.0003 | -8.0224 |
| Zinc finger protein COL 6 | - | 83.6 | Q8LG76 | *A. thaliana* | 94 | 0 | 11.1586 | -7.80201 |
| XTH-7 | - | 119 | Q8LER3 | *A. thaliana* | 84 | 0 | 9.5335 | -7.57493 |
| PUP4 | - | 65.9 | C0PT89 | *P. sitchensis* | 58 | 0 | 7.6918 | -7.26525 |
| E3 ubiquitin-protein ligase RHA2A | b2 or 5 or 6 | 73.2 or 73.2 or 73.2 | Q9ZT50 | *A. thaliana* | 119 | 0.3222 | 20.9088 | -6.02001 |
| PUP5 | 1 or 4 | 95.9 or 63.9 | B8LN73 | *P. sitchensis* | 81 | 0.1611 | 9.8585 | -5.93534 |
| Geranyl diphosphate synthase | - | 57 | Q8LKJ1 | *Abies grandis* | 82 | 0.1611 | 9.7502 | -5.9194 |

GolS=galactinol synthase, PUP=putative uncharacterized protein, PDR=probable disease resistance, XTH=probable xyloglucan endotransglucosylase/hydrolase.

Table S9. Reference genes from published conifer studies with their forward and reverse primer sequences.

| **Gene name** | **Primer forward (5'→3')** | **Primer reverse (5'→3')** | **Source** | **Species** |
| --- | --- | --- | --- | --- |
| 18S rRNA | CGGCGGATGTTGCTCTAAG | TCTGTCAATCCTTACTATGTCTGG | Phillips *et al.* (2009) | *Picea abies* |
| 18S rRNA* | TTCTGCCCTATCAACTTTCG | GATGTGGTAGCCGTTTCTCA | Gonçalves *et al.* (2005) | *Pinus pinaster* |
| α-tubulin | GGCATACCGGCAGCTCTTC | AAGTTGTTGGCGGCGTCTT | Fossdal *et al.* (2007) from Hietala *et al.* (2004) | *Picea abies* |
| eIF4A2 | ATTCAGGTGGGTGTTTTCTCT | GTGTGATTGCCAGGGTCTC | Gonçalves *et al.* (2005) | *Pinus pinaster* |
| eIF4A2 | AGTAAGCCCGTGAGGATTC | AGTCAGCCAGTCAACCTTTC | Palovaara & Hakman (2008) | *Picea abies* |
| GAPDH | AGGAGAGGACCAAGATAGGAAT | CCGCTGATGGAGCAGAAAT | Gonçalves *et al.* (2005) | *Pinus pinaster* |
| GAPDH | GGTTGCTTGTGGACCTTTAGC | CTGGGTTGCCTTTTGCTTTC | Palovaara & Hakman (2008) | *Picea abies* |
| TIF5A | GTGCCATCTTCACACAACTGC | CAGATTCAGTCAGCAGGCTAAC | Ralph *et al.* (2007) | *Picea spp.* |
| β-tubulin | CGTTACCTGCTGCCTGAG | GCTCTGTATTGCTGTGAACC | Phillips *et al.* (2009) | *Picea abies* |
| Ubiquitin | CGGCAAGCAGTTGGAGGATGG | CGGAGGACGAGGTGAAGAGTGG | Phillips *et al.* (2009) | *Picea abies* |
| Ubiquitin | GATTTATTTCATTGGCAGGC | AGGATCATCAGGATTTGGGT | Gonçalves *et al.* (2005) | *Pinus pinaster* |
| Ubiquitin conjugating enzyme 1 | GGAACAGTGGAGTCCTGCTT | CCTTGCGGTGGACTCATATT | Palovaara & Hakman (2008) | *Picea abies* |

*used for normalization

**Table S10.** **Ranking of the reference genes according to their expression stability using geNorm and Normfinder with their M-value or inter-group and average intra-group variability in brackets.**

| **Reference gene** | **geNorm (M-value)** | **Normfinder (inter-; intra-group variability)** |
| --- | --- | --- |
| *TPC1* | 1 (0.72) | 1 (0.216; 0.152) |
| *18S rRNA* | 1 (0.72) | 2 (0.221; 0.155) |
| *GAPDH* | 2 (1.02) | 4 (0.797; 0.726) |
| *elF4A2* | 3 (1.34) | 3 (0.791; 1.512) |

**Table S11. Amplification-efficiencies (E=[0, 1] and standard deviations (SD) calculated according to Liu & Saint [7].**

| **Gene** | **Efficiency** | **SD** |
| --- | --- | --- |
| *XTH7* | 0.61 | 0.07 |
| *BAM1(b)* | 0.72 | 0.05 |
| *ERD10* | 0.64 | 0.05 |
| *PUP1(a)* | 0.74 | 0.06 |
| *TPC1* | 0.71 | 0.05 |
| *elF4A2* | 0.67 | 0.05 |
| *GAPDH* | 0.54 | 0.06 |
| 18S rRNA | 0.77 | 0.03 |

**References**

1. Phillips MA, D’Auria JC, Luck K, Gershenzon J. Evaluation of Candidate Reference Genes for Real-Time Quantitative PCR of Plant Samples Using Purified cDNA as Template. Plant Mol Biol Report. 2009;27: 407–416. doi:10.1007/s11105-008-0072-1

2. Gonçalves S, Cairney J, Maroco J, Oliveira MM, Miguel C. Evaluation of control transcripts in real-time RT-PCR expression analysis during maritime pine embryogenesis. Planta. 2005;222: 556–563. doi:10.1007/s00425-005-1562-0

3. Fossdal CG, Nagy NE, Johnsen Ø, Dalen LS. Local and systemic stress responses in Norway spruce: Similarities in gene expression between a compatible pathogen interaction and drought stress. Physiol Mol Plant Pathol. 2007;70: 161–173. doi:10.1016/j.pmpp.2007.09.002

4. Hietala AM, Kvaalen H, Schmidt A, Jøhnk N, Solheim H, Fossdal CG. Temporal and Spatial Profiles of Chitinase Expression by Norway Spruce in Response to Bark Colonization by Heterobasidion annosum. Appl Environ Microbiol. 2004;70: 3948–3953. doi:10.1128/AEM.70.7.3948-3953.2004

5. Palovaara J, Hakman I. Conifer WOX-related homeodomain transcription factors, developmental consideration and expression dynamic of WOX2 during Picea abies somatic embryogenesis. Plant Mol Biol. 2008;66: 533–549. doi:10.1007/s11103-008-9289-5

6. Ralph SG, Jancsik S, Bohlmann J. Dirigent proteins in conifer defense II: Extended gene discovery, phylogeny, and constitutive and stress-induced gene expression in spruce (Picea spp.). Phytochemistry. 2007;68: 1975–1991. doi:10.1016/j.phytochem.2007.04.042

7. Liu W, Saint DA. A New Quantitative Method of Real Time Reverse Transcription Polymerase Chain Reaction Assay Based on Simulation of Polymerase Chain Reaction Kinetics. Anal Biochem. 2002;302: 52–59. doi:10.1006/abio.2001.5530
